# The Significance of Comprehensive Metabolic Phenotypes in Cancer Risk: A Japan Multi-Institutional Collaborative Cohort Study

**DOI:** 10.1158/2767-9764.CRC-24-0249

**Published:** 2024-11-21

**Authors:** Takeshi Watanabe, Tien Van Nguyen, Sakurako Katsuura-Kamano, Kokichi Arisawa, Masashi Ishizu, Taichi Unohara, Keitaro Tanaka, Chisato Shimanoe, Mako Nagayoshi, Takashi Tamura, Yuko Kubo, Yasufumi Kato, Isao Oze, Hidemi Ito, Nobuaki Michihata, Yohko Nakamura, Shiroh Tanoue, Chihaya Koriyama, Sadao Suzuki, Hiroko Nakagawa-Senda, Teruhide Koyama, Satomi Tomida, Kiyonori Kuriki, Naoyuki Takashima, Akiko Harada, Kenji Wakai, Keitaro Matsuo, for the J-MICC Study Group

**Affiliations:** 1 Department of Preventive Medicine, Tokushima University Graduate School of Biomedical Sciences, Tokushima, Japan.; 2 Thai Binh University of Medicine and Pharmacy, Thai Binh, Vietnam.; 3 Department of Food Nutritional Science, Tokushima Bunri University, Tokushima, Japan.; 4 Student Lab, Tokushima University Faculty of Medicine, Tokushima, Japan.; 5 Department of Preventive Medicine, Faculty of Medicine, Saga University, Saga, Japan.; 6 Department of Pharmacy, Saga University Hospital, Saga, Japan.; 7 Department of Preventive Medicine, Nagoya University Graduate School of Medicine, Nagoya, Japan.; 8 Division of Cancer Epidemiology and Prevention, Aichi Cancer Center Research Institute, Nagoya, Japan.; 9 Division of Cancer Information and Control, Aichi Cancer Center Research Institute, Nagoya, Japan.; 10 Department of Descriptive Cancer Epidemiology, Nagoya University Graduate School of Medicine, Nagoya, Japan.; 11 Cancer Prevention Center, Chiba Cancer Center Research Institute, Chiba, Japan.; 12 Department of Epidemiology and Preventive Medicine, Kagoshima University Graduate School of Medical and Dental Sciences, Kagoshima, Japan.; 13 Department of Public Health, Nagoya City University Graduate School of Medical Sciences, Nagoya, Japan.; 14 Department of Epidemiology for Community Health and Medicine, Kyoto Prefectural University of Medicine, Kyoto, Japan.; 15 Department of Endocrine and Breast Surgery, Kyoto Prefectural University of Medicine, Kyoto, Japan.; 16 Laboratory of Public Health, Division of Nutritional Sciences, School of Food and Nutritional Sciences, University of Shizuoka, Shizuoka, Japan; 17 NCD Epidemiology Research Center, Shiga University of Medical Science, Otsu, Japan.; 18 Department of Cancer Epidemiology, Nagoya University Graduate School of Medicine, Nagoya, Japan.

## Abstract

The present study investigated the relationship between metabolic phenotypes and the risk of cancer in a Japanese population using the criteria of metabolic phenotypes based on an examination and those based on questionnaires. We used data from 25,357 subjects for examination-based analyses and those from 53,042 subjects for questionnaire-based analyses in the Japan Multi-institutional Collaborative Cohort study. Metabolic phenotypes were defined by classifying subjects according to their body mass index (BMI; obesity: BMI  ≥25 kg/m^2^; normal weight: BMI  <25 kg/m^2^) and the number of metabolic abnormalities. Metabolic abnormalities were defined according to metabolic syndrome components of the Joint Interim Statement criteria for examination-based analyses and self-reported histories of diabetes, dyslipidemia, and hypertension for questionnaire-based analyses. Cox proportional hazards regression analyses adjusted for potential confounders were performed for total and site-specific cancer incidence rates according to metabolic phenotypes. Metabolically unhealthy obesity (MUHO) was significantly associated with cancer incidence in both examination-based [HR (95% confidence interval), 1.17 (1.01–1.36)] and questionnaire-based analyses [HR (95% confidence interval), 1.15 (1.04–1.26)]. Regarding site-specific cancer in questionnaire-based analyses, metabolically healthy obesity and MUHO were associated with colorectum and liver cancers in all subjects and with breast cancer in female subjects. Subjects with a metabolically unhealthy normal weight had a higher risk of pancreatic cancer. Moreover, MUHO was associated with corpus uteri cancer in female subjects. This prospective cohort study suggests that metabolic phenotypes are important risk factors for total and some site-specific cancers in Japanese adults.

**Significance:**

The prospective cohort study in a large Japanese population suggested that metabolic phenotypes are important risk factors for total and some site-specific cancers in Japanese adults. Moreover, the risk of each site-specific cancer may differ according to metabolic phenotypes.

## Introduction

Obesity is a serious public health issue worldwide (1), and the number of people living with obesity is increasing both globally (2) and in Japan (3). Obesity and other cardiovascular risks, i.e., hypertension, hyperglycemia, and dyslipidemia, form the complex called metabolic syndrome (MetS; refs. 4–7). Although obesity and other metabolic abnormalities have been identified as independent risk factors for cardiovascular disease (CVD), the incidence and mortality of diseases including CVD may differ depending on their combination (8, 9). For example, a previous study showed that metabolically unhealthy normal weight (MUNW) and metabolically unhealthy obese (MUHO), but not metabolically healthy obese (MHO), subjects had a higher risk of CVD and all-cause mortality than metabolically healthy normal weight (MHNW) subjects (8). Furthermore, the risk of diseases such as atherosclerotic CVD was found to be higher in MUNW, MUHO, and MHO subjects than in MHNW subjects (9). The categorization of subjects based on obesity and the metabolic health status is called the metabolic phenotype and has attracted attention (1, 10–12). The pathogenesis of metabolic abnormalities has also been suggested to differ between obese and normal weight subjects, such as the underlying genetic background (13). Therefore, assessments of differences in the risk of various diseases based on metabolic phenotypes, not simple obesity or MetS, may contribute to the prevention of diseases according to patient characteristics.

Similar to CVD, the relationship between obesity, MetS, and cancer has been well documented. For example, an umbrella review of systematic reviews and meta-analyses showed that the relationship between adiposity and 11 cancers, including colon, breast, and pancreatic cancers, was supported by strong evidence (14). The relationship between MetS and site-specific cancers has been extensively investigated (15). A meta-analysis of 43 studies revealed that MetS was significantly associated with various cancers, including liver, colorectal, and breast cancers (15). On the other hand, evidence for the relationship between metabolic phenotypes and cancer is limited. A prospective cohort study in Sweden showed that obese subjects regardless of metabolic health had a higher risk of total cancer than MHNW subjects (16). In a prospective cohort study conducted in Taiwan, metabolically unhealthy overweight, but not obese, subjects had a significantly higher total cancer risk than MHNW subjects (17). Recent studies performed in Europe reported a relationship between metabolic phenotypes and obesity-related site-specific cancer (18, 19). In Japan, MUHO was associated with total cancer mortality (20). Although data from anthropometric and blood examinations are necessary to estimate metabolic phenotypes, they are costly and time consuming; therefore, criteria by which metabolic phenotypes may be classified based simply on information from questionnaires may be useful for future epidemiologic studies.

The present study investigated the relationships between metabolic phenotypes and total and site-specific cancer incidence rates using both examination- and questionnaire-based analyses of a large Japanese population.

## Materials and Methods

### Study design and subjects

A prospective cohort analysis was conducted using data from the Japan Multi-institutional Collaborative Cohort (J-MICC) study. Details on the J-MICC study have previously been reported (21–23). Briefly, the J-MICC study was launched in April 2005 and recruited subjects of ages 35 to 69 years from 14 research areas in Japan. The main purpose of the J-MICC study was to confirm the interactions of lifestyle and genetic factors with the risk of chronic diseases. The study protocol was approved by the Ethics Committee of Aichi Cancer Center Research Institute (No. H2210001A), Tokushima University Hospital (No. 466-15), and all other institutions participating in the J-MICC Study. Written informed consent was obtained from all subjects.

**Figure 1:**
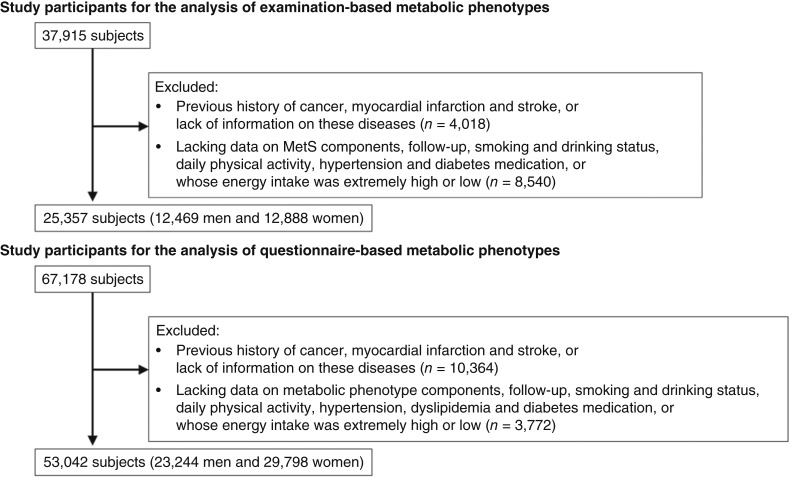
Flowcharts of the selection of study participants. **A,** Study participants for analyses of examination-based metabolic phenotypes. **B,** Study participants for analyses of questionnaire-based metabolic phenotypes.

We selected study subjects from the participants of the J-MICC study for examination- and questionnaire-based analyses. Examination- and questionnaire-based analyses were different in the definition of metabolic phenotypes. Metabolic phenotypes in examination-based analyses were classified according to anthropometric and biological data, and those in questionnaire-based analyses were classified according to the self-reported medical history from the questionnaire. These definitions are described in detail in the “Definitions of MetS and metabolic phenotypes” section. Dataset version 20210901 was used. In examination-based analyses, 37,915 individuals (17,561 men and 20,354 women) from seven sites that used the same questionnaire and conducted the blood examination needed to diagnose MetS (Okazaki, Shizuoka, Takashima, Kyoto, Kagoshima, Tokushima, and Shizuoka-Sakuragaoka) were initially included. We excluded subjects with a history of cancer, myocardial infarction, or stroke or missing information on these diseases (*n* = 4,018), with missing data on the follow-up period (*n* = 2), with missing data on smoking and drinking habits or physical activity or whose total energy intake was extremely high or low (>4,000 or ≤1,000 kcal, *n* = 2,316), or with missing data on the BMI, systolic blood pressure (SBP), diastolic blood pressure (DBP), triglycerides, High-density lipoprotein (HDL) cholesterol, or fasting blood glucose (*n* = 6,222). Therefore, 25,357 subjects (12,469 men and 12,888 women) were ultimately included. In questionnaire-based analyses, 67,178 individuals (29,852 men and 37,326 women) from 11 sites that used the same questionnaire (Chiba, Aichi Cancer Center, Okazaki, Shizuoka, Daiko, Takashima, Kyoto, Saga, Kagoshima, Tokushima, and Shizuoka-Sakuragaoka) were initially included. We excluded subjects with a history of cancer, myocardial infarction, or stroke or missing information on these diseases (*n* = 10,364), with missing data on the follow-up period (*n* = 41), with missing data on smoking and drinking habits or physical activity or whose total energy intake was extremely high or low (*n* = 3,092), or with missing data on the self-reported history of hypertension, dyslipidemia, and diabetes (*n* = 639). Therefore, 53,042 subjects (23,244 men and 29,798 women) were ultimately included. Both selections of study subjects are shown in Fig. 1.

### Questionnaire

Data collection was performed based on a structured self-administered questionnaire, which subjects completed, and the data obtained were checked by trained staff at the survey. The questionnaire consisted of a series of questions about subjects’ sociodemographic characteristics, lifestyle, medical history, and medications. Dietary intakes of green and yellow vegetables, light-colored vegetables, fruit, and miso soup were assessed using a validated, short food frequency questionnaire (24, 25). Total energy and the intake of 26 nutrients, including calcium, was assessed with the program developed and validated at the Department of Public Health, Nagoya City University School of Medicine (26). Dietary intakes of green and yellow vegetables, light-colored vegetables, fruit, miso soup, and calcium were log-transformed and energy-adjusted using the residual method. Dietary vegetable intake was calculated by adding the intake of green and yellow vegetables and light-colored vegetables.

Educational levels were classified into four categories (≤9 years, 10–15 years, ≥16 years, and unknown). Smoking habits were classified into three categories (current, ex, and non), and the average number of cigarettes per day and age at the initiation of habitual smoking were noted. Pack-years were calculated by multiplying the average number of cigarettes per day by the number of years smoked and divided by 20 (one pack). Drinking habits were classified into three categories (current, ex, and non), and the frequency and amount consumed each time for the following six alcoholic drinks were noted: Japanese sake, shochu, shochu-based cocktails, beer, whiskey, and wine. Ethanol intake (g/day) by current drinkers was calculated based on the amount of ethanol present in each alcoholic drink. Total physical activity during leisure time was estimated using a questionnaire. The frequency (five categories from never to ≥5 times/week) and average duration (six categories from ≤30 minutes to ≥4 hours) of the following three groups was reported by subjects: light-intensity exercise (e.g., walking and golf) at 3.4 metabolic equivalent of tasks (METs), moderate-intensity exercise (e.g., jogging and swimming) at 7.0 METs, and vigorous-intensity exercise (e.g., marathon running) at 10.0 METs. The three levels of leisure-time physical activity were calculated as MET hours/week (MET level  ×  hours of activity  ×  events per week), and these values were summed and used as the value for total physical activity in the present study.

### Anthropometric and biochemical measurements

Height (cm), weight (kg), SBP and DBP, serum triglycerides, HDL cholesterol, and blood glucose were measured at each research site according to standardized protocols. BMI (kg/m^2^) was calculated as weight (kg) divided by the square of height (m^2^).

### Definitions of MetS and metabolic phenotypes

The definitions of MetS and metabolic phenotypes were described in a previous study (27). Briefly, we defined MetS based on the Joint Interim Statement criteria (28). BMI (≥25 kg/m^2^) was used instead of waist circumference (WC for Asians, including Japanese: ≥90 for men and ≥80 for women) because WC was not measured in all subjects (28, 29). MetS was defined as the combined presence of at least three of the following five criteria: (i) obesity: BMI ≥25 kg/m^2^; (ii) elevated blood pressure: SBP ≥130 mmHg and/or DBP ≥85 mmHg and/or the self-reported use of antihypertensive drugs; (iii) serum triglyceride level ≥150 mg/dL; (iv) serum HDL cholesterol level <40 mg/dL for men and <50 mg/dL for women; and (v) blood glucose level  ≥100 mg/dL and/or the self-reported use of antidiabetic drugs.

In the classification of metabolic phenotypes, subjects were categorized into four groups based on the BMI (normal weight or obesity) and the metabolic health status (healthy or unhealthy). Examination-based metabolic phenotypes were defined using data from anthropometric and blood examinations. Subjects with a normal weight (BMI <25 kg/m^2^) were divided into two phenotypes: MUNW and MHNW (≥1 or no components of MetS, respectively). Subjects with obesity (BMI ≥25 kg/m^2^) were classified as MUHO and MHO (≥1 or no components of MetS other than BMI, respectively). Questionnaire-based metabolic phenotypes were defined using the BMI calculated from self-reported height and weight, a history of hypertension, dyslipidemia, or diabetes, and a medication history for these morbidities. Subjects stratified by obesity with ≥1 disease from the self-reported history or medication for hypertension, dyslipidemia, or diabetes were categorized as metabolically unhealthy. Sensitivity analyses changing the cut-off were also conducted in both examination- and questionnaire-based metabolic phenotypes.

### Follow-up and cancer ascertainment

Information on cancer incidence was collected through national cancer registries, regional cancer registries, patient notifications from hospitals, and reports from subjects confirmed by medical records. Data from the national cancer registries provided to us according to the Cancer Registry Promotion Act were processed and analyzed independently for this study. All cancer cases were classified according to the International Classification of Diseases, 10th revision. The outcome of the present study was the incidence of total cancer (C001–809), stomach cancer (C16), colon and rectum cancers (C18–21), liver cancer (C22), pancreatic cancer (C25), and lung and bronchus cancers (C34) in all subjects, breast cancer (C50) and corpus uteri cancer (C54) in women, and prostate cancer in men (C61). Moreover, colorectum cancer was separated into proximal colon cancer (C18.0–18.4), distal colon cancer (C18.5–18.7), and rectum cancer (C19–C20). In analyses of cancer incidence, person-years of follow-up were calculated using the time from the date of the baseline survey until the occurrence of cancer, death, moving, or the end of the follow-up period (December 31, 2021). Cancer incidence was calculated using the number of incidences divided by the person-years of follow-up. During a median (25%, 75%) follow-up of 8.0 (5.5, 10.2) years, 1,584 (951 men and 633 women) cancer cases were identified in subjects in examination-based analyses, whereas there were 4,467 (2,423 men and 2,044 women) cancer cases in subjects in questionnaire-based analyses during a median (25%, 75%) follow-up of 9.1 (5.9, 10.5) years.

### Statistical analysis

Regarding the baseline characteristics of subjects according to the obesity status, the χ^2^ test for categorical variables and the Wilcoxon rank-sum test for continuous variables were applied. Multivariable Cox proportional hazards regression analyses were conducted to assess the relationships between MetS, the number of components, each individual component, and the incidence of cancer. The relationship between metabolic phenotypes and cancer incidence was also examined using MHNW subjects as a reference. Model 1 was adjusted for age (continuous), sex, research sites (7 categories in examination-based analyses and 11 categories in questionnaire-based analyses), and educational background (four categories: ≤9 years, 10–15 years, ≥16 years, and unknown). Model 2 was additionally adjusted for pack-years (four categories: 0, >0 and <20, ≤20, and unknown) and the drinking status (four categories: never, ex, >0 and <20 g/day, and ≥20 g/day), and physical activity levels (quartiles). Model 3 was adjusted for energy-adjusted vegetable, fruit, and miso soup intakes (quartiles). Model 4 was additionally adjusted for hormone replacement therapy, age of menarche (four categories: <11, ≥11 and <15, ≥15, and unknown), and menopausal status and age of menopause (four categories: premenopause, <55, ≥55, and unknown) in the analyses of breast cancer. Moreover, in addition breast cancer analyses, model 4 was adjusted for history of ovarian disease (three categories: non, current, and past) in the analyses of corpus uteri cancer. Antipyretic use (two categories: yes or no), calcium intake (quartiles), and red and processed meat intake (quartiles) were additionally adjusted in model 4 in the analyses of colorectum cancer. History of hepatitis B and C (two categories: yes or no) were adjusted in model 4 in the analyses of liver cancer. We conducted sensitivity analyses by handling subjects who had cancer within 1 year as censored. We additionally conducted analyses by handling subjects who had cancer within 2 years as censored in the analyses of total and site-specific cancers with the exception of cancers which were fewer than 10 cases in some groups of metabolic phenotypes. The test for trends in the relationship between the number of components of MetS or metabolic phenotypes and cancer incidence was performed using a likelihood ratio test. Proportional hazards assumptions were checked for each variable using the Schoenfeld residual method. The results obtained indicated that these assumptions were not violated over time.

All statistical analyses were performed using Statistical Analysis System (SAS) statistical software (version 9.4 for Windows; SAS Institute Inc., RRID: SCR_008567). Statistical tests were based on two-sided probabilities, and *P* values < 0.05 were considered significant. Forest plot was created using the forestploter package of R (version 4.3.1, RRID: SCR_001905).

### Data availability

The anonymized minimum data needed to replicate the results of the present study are available upon reasonable request to the corresponding author and after approval by all the participating institutions, the Ministry of Health, Labor, and Welfare, and the National Cancer Registry, Japan.

## Results

The baseline characteristics of subjects according to obesity for examination- and questionnaire-based analyses are shown in Table 1. Among 25,357 subjects in examination-based analyses, 6,309 (24.9%) were obese (3,812 men and 2,497 women). Among 53,037 subjects in questionnaire-based analyses, 11,559 (21.8%) were obese (6,727 men and 4,832 women). Among subjects in examination-based analyses, obese subjects were more likely to be men and less physically active. Furthermore, obese subjects had a shorter duration of education and were more likely to be current smokers, smoke more cigarettes, be current drinkers, and drink more alcohol. Obese subjects also had significantly more self-reported medical histories of colorectal polyps, fatty liver, high blood pressure, diabetes, and dyslipidemia but a lower medical history of chronic gastritis. In addition, obese subjects were taking more medications for hypertension, diabetes, and high blood cholesterol but less for constipation. The overall results were similar between subjects in examination-based and questionnaire-based analyses; however, in questionnaire-based analyses, obese subjects were slightly older and had a higher self-reported medical history of hepatitis B. Sex-stratified analyses were shown in Supplementary Table S1. Obese female subjects were more likely to be postmenopausal women. Although the overall results were similar between male and female subjects, the significant difference in the duration of education and antipyretic medication between normal weight and obesity subjects were only observed in female subjects, and significant difference of medical history of hepatitis B was only observed in male subjects (Supplementary Table S1).

**Table 1:** Background characteristics of participants according to obesity classification

Subjects for the analysis of examination-based metabolic phenotypes				Subjects for the analysis of questionnaire-based metabolic phenotypes			
Characteristics^a^	Normal weight	Obesity	*P* value^b^	Characteristics^a^	Normal weight	Obesity	*P* value^b^
	(*n* = 19,048)	(*n* = 6,309)			(*n* = 41,483)	(*n* = 11,559)	
Age (years)	56 (46, 63)	56 (47, 63)	0.136	Age (years)	55 (46, 62)	55 (47, 62)	<0.0001
Exercise during leisure time (MET hours/week)	6.0 (0.4, 17.9)	5.1 (0, 17.9)	<0.0001	Exercise during leisure time (MET hours/week)	6.0 (0.4, 17.9)	5.1 (0.4, 16.2)	<0.0001
Sex				Sex			
Men	8,657 (45.4)	3,812 (60.4)	<0.0001	Men	16,517 (39.8)	6,727 (58.2)	<0.0001
Women	10,391 (54.6)	2,497 (39.6)	—	Women	24,966 (60.2)	4,832 (41.8)	—
Educational background (years)				Educational background (years)			
≤9	2,240 (11.8)	1,002 (15.9)	<0.0001	≤9	3,344 (8.1)	1,382 (12.0)	<0.0001
10–15	12,211 (64.1)	3,762 (59.6)	—	10–15	27,355 (65.9)	6,992 (60.5)	—
≥16	4,479 (23.5)	1,505 (23.9)	—	≥16	10,582 (25.5)	3,126 (27.0)	—
Unknown	118 (0.6)	40 (0.6)	—	Unknown	202 (0.5)	59 (0.5)	—
Smoking habit				Smoking habit			
Current	3,024 (15.9)	1,125 (17.8)	<0.0001	Current	6,795 (16.4)	2,306 (20.0)	<0.0001
Past	4,227 (22.2)	1,759 (27.9)	—	Past	8,527 (20.6)	3,147 (27.2)	—
Never	11,797 (61.9)	3,425 (54.3)	—	Never	26,161 (63.1)	6,106 (52.8)	—
Pack-years				Pack-years			
0	11,797 (61.9)	3,425 (54.3)	<0.0001	0	26,161 (63.1)	6,106 (52.8)	<0.0001
>0 and <20	3,176 (16.7)	989 (15.7)	—	>0 and <20	6,694 (16.1)	1,825 (15.8)	—
≥20	3,693 (19.4)	1,744 (27.6)	—	≥20	7,963 (19.2)	3,388 (29.3)	—
Unknown	382 (2.0)	151 (2.4)	—	Unknown	665 (1.6)	240 (2.1)	—
Alcohol drinking				Alcohol drinking			
Never	7,940 (41.7)	2,433 (38.6)	<0.0001	Never	17,261 (41.6)	4,507 (39.0)	<0.0001
Past	308 (1.6)	106 (1.7)	—	Past	848 (2.0)	248 (2.2)	—
>0 and <20 g/day	6,585 (34.6)	1,969 (31.2)	—	>0 and <20 g/day	14,683 (35.4)	3,600 (31.1)	—
≥20 g/day	4,215 (22.1)	1,801 (28.6)	—	≥20 g/day	8,691 (21.0)	3,204 (27.7)	—
Medical history				Medical history			
Gastric ulcer	2,313 (12.1)	694 (11.0)	0.015	Gastric ulcer	5,534 (13.3)	1,485 (12.9)	0.166
Chronic gastritis	2,247 (11.8)	532 (8.4)	<0.0001	Chronic gastritis	5,301 (12.8)	1,179 (10.2)	<0.0001
Colorectal polyps	1,648 (8.7)	623 (9.9)	0.003	Colorectal polyps	3,340 (8.1)	1,135 (9.8)	<0.0001
Hepatitis B	230 (1.2)	92 (1.5)	0.123	Hepatitis B	496 (1.2)	178 (1.5)	0.004
Hepatitis C	156 (0.8)	53 (0.8)	0.872	Hepatitis C	442 (1.1)	143 (1.2)	0.118
Fatty liver	1,159 (6.1)	1,172 (18.6)	<0.0001	Fatty liver	2,539 (6.1)	2,309 (20.0)	<0.0001
Asthma	1,156 (6.1)	398 (6.3)	0.493	Asthma	2,600 (6.3)	772 (6.7)	0.109
High blood pressure	2,916 (15.3)	1,908 (30.2)	<0.0001	High blood pressure	5,680 (14.1)	3,457 (29.9)	<0.0001
Diabetes	835 (4.4)	544 (8.6)	<0.0001	Diabetes	1,760 (4.2)	970 (8.4)	<0.0001
Dyslipidemia	2,621 (13.8)	1,229 (19.5)	<0.0001	Dyslipidemia	5,919 (14.3)	2,443 (21.1)	<0.0001
Medication				Medication			
High blood pressure	2,389 (12.5)	1,683 (26.7)	<0.0001	High blood pressure	4,681 (11.3)	2,974 (25.7)	<0.0001
Diabetes	504 (2.7)	393 (6.2)	<0.0001	Diabetes	1,071 (2.6)	713 (6.2)	<0.0001
High blood cholesterol	1,527 (8.0)	793 (12.6)	<0.0001	High blood cholesterol	3,115 (7.5)	1,458 (12.6)	<0.0001
Sleeping pills	661 (3.5)	221 (3.5)	0.902	Sleeping pills	1,696 (4.1)	431 (3.7)	0.081
Antipyretic	548 (2.9)	197 (3.1)	0.317	Antipyretic	1,282 (3.1)	395 (3.4)	0.076
Laxative	766 (4.0)	193 (3.1)	0.0005	Laxative	1,924 (4.6)	377 (3.3)	<0.0001

a Median (25%, 75%) or number of subjects (%).

b Wilcoxon’s rank-sum test or χ^2^ test.

Supplementary Table S2 shows the HR [95% confidence interval (CI)] for the risk of cancer according to MetS, the number of components, or each component. Subjects with MetS had a higher risk of cancer than those without MetS in all models. The number of MetS components was associated with cancer incidence. Among MetS components, marginally significant association of obesity, hypertension, and elevated blood glucose with a higher risk of cancer were observed. We obtained similar results in examination- and questionnaire-based analyses. Among the components examined, i.e., obesity, hypertension, dyslipidemia, and diabetes, in the self-administered questionnaire, obesity, hypertension, and diabetes were associated with a higher risk of cancer. The results obtained on the relationship between the number of components and cancer incidence in subjects stratified by obesity are shown in Supplementary Table S3. In examination- and questionnaire-based criteria, the number of components was associated with cancer incidence in obese subjects only (examination-based analyses, model 3, *P*_trend_ = 0.009; questionnaire-based analyses, model 3, *P*_trend_ = 0.041). Sex-stratified analyses were showed in Supplementary Table S4. The association between the number of components and cancer incidence in obese subjects was only observed in male subjects both in examination- and questionnaire-based criteria (Supplementary Table S4). Supplementary Table S5 shows the relationships between individual components and cancer incidence in subjects stratified by obesity. In examination-based analyses, an elevated blood glucose level was associated with cancer in obese subjects only [model 3, HR (95% CI), 1.30 (1.07–1.58)]. In questionnaire-based analyses, high blood pressure and diabetes were associated with cancer incidence in both normal and obese subjects; however, point estimates were higher in obese subjects. Sex-stratified analyses were also conducted (Supplementary Table S6). Hypertension was significantly associated with cancer incidence in male obese subjects in questionnaire-based analyses and in female normal weight subjects in both analyses (Supplementary Table S6). The same analyses as those shown in Supplementary Tables S3 and S5 by handling subjects with cancer within 1 year or 2 years as censored were conducted, and similar results were obtained (Supplementary Tables S7 and S8).

**Figure 2:**
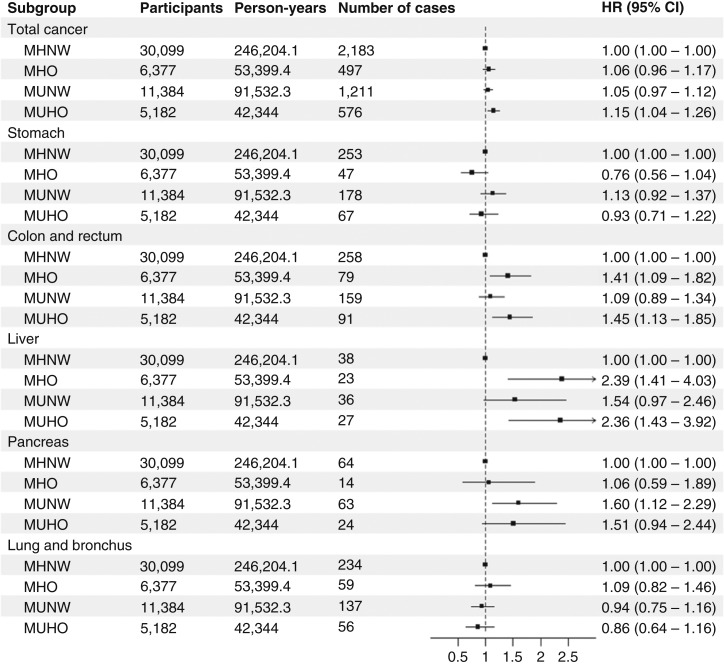
Relationships between questionnaire-based metabolic phenotypes and site-specific cancers. HRs and 95% CIs are shown as points and error bars. Cox proportional hazard models to estimate association between metabolic phenotypes and site-specific cancers after adjusting for age, sex, research sites, educational background, pack-years, drinking habit, physical activity level, and miso soup, fruit, and vegetable consumption.

**Figure 3:**
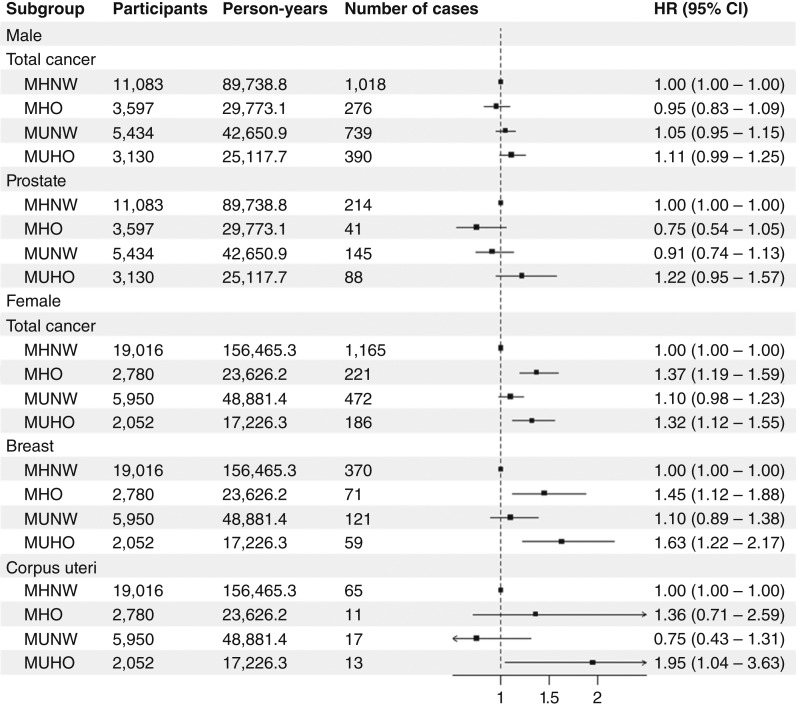
Sex-stratified analyses of relationships between questionnaire-based metabolic phenotypes and site-specific cancers. HRs and 95% CIs are shown as points and error bars. Sex-stratified Cox proportional hazard models to estimate association between metabolic phenotypes and site-specific cancers after adjusting for age, research sites, educational background, pack-years, drinking habit, physical activity level, and miso soup, fruit, and vegetable consumption.

**Figure 4:**
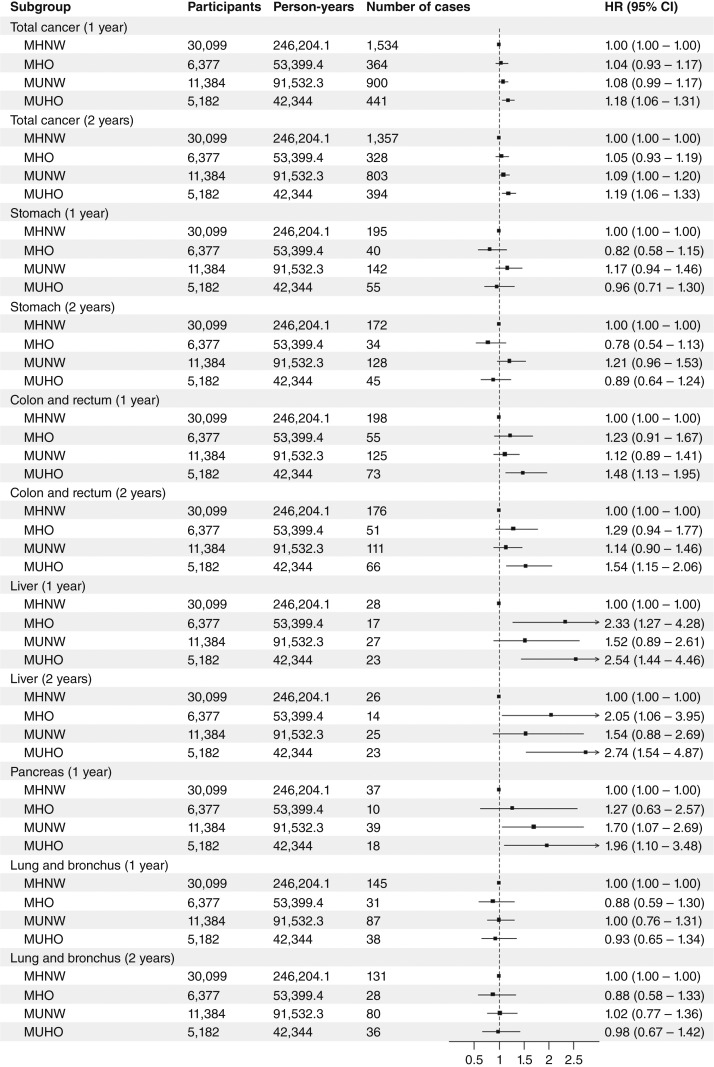
Relationships between questionnaire-based metabolic phenotypes and site-specific cancers handling subjects who had cancer within 1 year or 2 years as censored. HRs and 95% CIs are shown as points and error bars. Cox proportional hazard models to estimate association between metabolic phenotypes and site-specific cancers after adjusting for age, sex, research sites, educational background, pack-years, drinking habit, physical activity level, and miso soup, fruit, and vegetable consumption.

**Figure 5:**
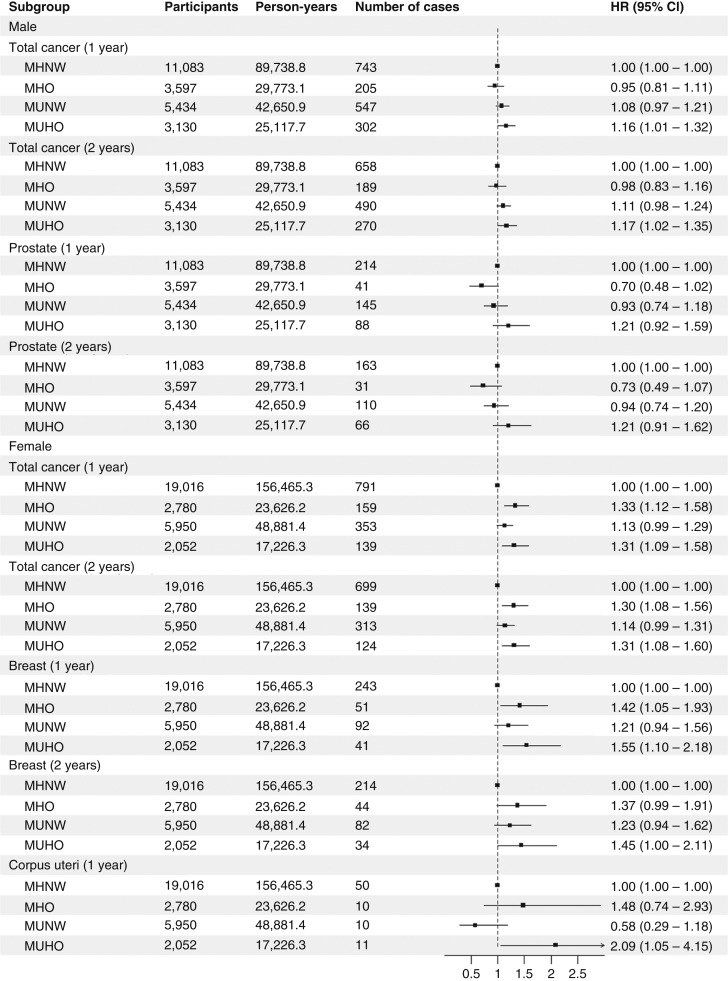
Sex-stratified analyses of relationships between questionnaire-based metabolic phenotypes and site-specific cancers handling subjects who had cancer within 1 year or 2 years as censored. HRs and 95% CIs are shown as points and error bars. Sex-stratified Cox proportional hazard models to estimate association between metabolic phenotypes and site-specific cancers after adjusting for age, research sites, educational background, pack-years, drinking habit, physical activity level, and miso soup, fruit, and vegetable consumption.

Figures 2 and 3 and Supplementary Table S9 show the relationship between metabolic phenotypes and cancer incidence in questionnaire-based analyses. MUHO was associated with total cancer in both examination-based analyses [model 3, HR (95% CI), 1.17 (1.01–1.36)] and questionnaire-based analyses [model 3, HR (95% CI), 1.15 (1.04–1.26); Supplementary Table S9]. Analyses of site-specific cancer and sex-stratified analyses were also conducted using questionnaire-based criteria. MHO and MUHO were associated with colorectal cancer and liver cancer in all subjects (Fig. 2; Supplementary Table S9). MHO and MUHO were also associated with total cancer and breast cancer in female subjects (Fig. 3; Supplementary Table S9). Among female subjects, MUHO subjects had a higher risk of corpus uteri cancer (Fig. 3; Supplementary Table S9). Among all subjects, MUNW was associated with pancreatic cancer (Fig. 2; Supplementary Table S9). The number of components was significantly associated with pancreatic cancer in normal weight subjects, and among components, diabetes was associated with pancreatic cancer in normal weight subjects (Supplementary Table S10). In sensitivity analyses handling subjects with cancer within 1 year as censored, a significant association was not observed between MHO and colorectal cancer (Fig. 4; Supplementary Table S11), whereas MUHO was associated with pancreatic cancer (Fig. 4; Supplementary Table S11). MUHO was associated with total cancer incidence in male subjects (Fig. 5; Supplementary Table S11). Moreover, MUHO had tendency of higher risk of total cancer in examination-based analyses, although it was not significant (Supplementary Table S11). In analyses handling subjects with cancer within 2 years as censored, the results were not so altered (Figs. 4 and 5; Supplementary Table S11). Regarding breast cancer, age-stratified analyses were also conducted (Supplementary Table S12). MUHO in subjects who were 54 years or younger and MHO and MUHO in subjects who were older than 54 was associated with a higher risk of breast cancer (Supplementary Table S12). Moreover, regarding colorectum cancer, analyses by separating proximal and distal colon cancers and rectum cancer were also conducted (Supplementary Table S13). MHO was significantly associated with distal colon cancer. The breakdown of site-specific cancer incidence is shown in Supplementary Table S14. It was similar between the subjects of examination-based and questionnaire-based analyses (Supplementary Table S14).

## Discussion

This prospective cohort study assessed the relationships between metabolic phenotypes and cancer incidence both in examination- and questionnaire-based analyses. The results obtained from both analyses were similar in the characteristics of participants (Table 1), the association between the number of components and cancer incidence (Supplementary Table S3), the association between each component and cancer incidence (Supplementary Table S5), and the association between metabolic phenotypes and total cancer incidence (Supplementary Table S9). The results from both analyses were also similar in sex-stratified analyses and sensitivity analyses handling subjects who had cancer within 1 or 2 years as censored (Supplementary Tables S1, S4, S6–S9, and S11). Moreover, associations were examined between metabolic phenotypes and various site-specific cancers; however, results were only obtained from questionnaire-based analyses (Figs. 2 and 3; Supplementary Table S9).

To date, few studies have reported a relationship between metabolic phenotypes and various site-specific cancers. A previous study using data from UK Biobank showed that obesity was associated with some cancers, such as endometrial cancer, regardless of the metabolic health status, whereas other cancers, including colorectal cancer, were associated with MUHO (18). Moreover, a pooled study conducted in Europe reported a relationship between metabolic phenotypes and obesity-related cancers (19). To the best of our knowledge, the present study is the first to examine the relationships between metabolic phenotypes and total and site-specific cancers in Asia, where even though the prevalence of obesity is lower, its impact on health is likely to be greater than in Europe (30, 31).

Although most of the present results were consistent with the findings of previous studies performed in Europe, there were some differences. For example, the inverse relationship between MUHO and prostate cancer reported in a previous study using data from UK Biobank was not observed in the present study (Fig. 3; Supplementary Table S9; ref. 18). Some studies indicated that MetS and obesity reduced PSA concentrations, which delayed the diagnosis of low-grade prostate cancer (32, 33). This may be one of the reasons for the inverse relationships observed between MUHO and prostate cancer in the previous study (18). On the other hand, severe obesity is less common in Japan, which may have contributed to the lack of relationship between MUHO and prostate cancer in the present study (Fig. 3; Supplementary Table S9; ref. 34). Moreover, MUHO subjects had a higher risk of corpus uteri cancer in the present study (Fig. 3; Supplementary Table S9). In previous studies, MHO and MUHO were associated with endometrial cancer, which is of the same classification of International Classification of Diseases, 10th revision, as corpus uteri cancer (C54) in the present study (17, 18). The reasons for this discrepancy remain unknown; however, differences in the criteria of obesity between the present study (BMI ≥25) and studies conducted in Europe (BMI ≥30) may play a role.

MHO and MUHO were associated with colorectal and liver cancers in all subjects and breast cancer in female subjects; however, an association was not observed between MHO and colorectal cancer in sensitivity analyses handling subjects who had cancer within 1 year or 2 years as censored (Figs. 2 and 5; Supplementary Tables S9 and S11). Previous studies showed that subjects with obesity or MetS had a higher risk of colorectal cancer (35, 36). Regarding liver and breast cancers, a study conducted in Japan showed that MetS was associated with liver cancer in all subjects (37) and that female subjects with MetS or a high BMI had a higher risk of liver and breast cancers (37). Obesity is closely related to the prevalence and severity of nonalcoholic fatty liver disease (NAFLD), which is emerging as one of the major causes of hepatocellular carcinoma, the most common type of liver cancer (38, 39). Insulin resistance and hyperinsulinemia accompanied by NAFLD may be involved in liver tumorigenesis by activating intracellular signaling pathways, such as the PI3K/Akt/mTOR pathway (39). Moreover, NAFLD may affect other gastrointestinal cancers, such as colorectal cancer (40). Adipokines, proinflammatory cytokines, insulin, and insulin-like growth factor secreted by adipose tissues have been implicated in the development of colorectal cancer (41). Regarding breast cancer, especially postmenopausal breast cancer, increased estrogen production by adipose tissues and the promotion of estrogen receptor expression and transactivation have been suggested to promote its progression (42). Epidemiologic studies also suggested that obesity may be more critical factor for postmenopausal breast cancer than premenopausal breast cancer (43). In our study, obesity was associated with breast cancer regardless of metabolic health in subjects who were older than 54 at baseline, and almost all the onset of breast cancer in these subjects may be at postmenopause, whereas only MUHO was associated with breast cancer in subjects who were 54 years or younger (Supplementary Table S12). Based on the present results, previous findings, and plausible mechanisms, obesity itself and/or other metabolic abnormalities accompanied by obesity may play important roles in the pathogenesis of these cancers.

MUNW, but not MHO nor MUHO, was associated with pancreatic cancer, although MUHO was also associated with pancreatic cancer in sensitivity analyses handling subjects who had cancer within 1 year as censored (Figs. 2 and 4; Supplementary Tables S9 and S11). Among normal weight subjects, the number of components was significantly associated with pancreatic cancer and diabetes may be the most important factor (Supplementary Table S10). Excess body weight is a well-established risk factor for pancreatic cancer; however, in cohort studies, the association is often underestimated because of the weight loss accompanied by diabetes, which subjects with prediagnostic pancreatic cancer often experience (44, 45). This may be a reason why the association between MUHO and pancreatic cancer was not observed in the main analysis and was unmasked in the sensitivity analysis handling subjects who had cancer within 1 year as censored in the present study (Figs. 2 and 4; Supplementary Tables S9 and S11). In the sensitivity analysis, there was still a significant association between MUNW and pancreatic cancer (Fig. 4; Supplementary Table S11). Pancreatitis, which is induced by risk factors such as alcohol and smoking, may causes diabetes characterized by a reduced range or the reference range of the BMI (46, 47). Pancreatogenic diabetes (type 3c diabetes *mellitus*) may play a role in the association between MUNW and pancreatic cancer in the present study.

The large number of study subjects is a major strength of the present study. The large sample size made it possible to adjust for various potential confounders in the analyses. Moreover, data from the relatively new cohort may reflect the current condition of MetS, metabolic phenotypes, and cancer in Japan. In contrast, there are several limitations that need to be addressed. Due to the lack of data on WC, we used BMI to assess obesity in examination-based analyses. However, a strong correlation between WC and BMI was previously reported in a study with various ethnic groups, including Japanese (Pearson’s correlation coefficients 0.921 for Japanese men and 0.922 for Japanese women; refs. 29, 48, 49). Furthermore, the assessment of metabolic phenotypes used baseline data, and a status change was not monitored. Moreover, information on the lifestyle and background characteristics of subjects and the components of metabolic phenotypes in questionnaire-based analyses were based on a self-reported questionnaire; therefore, misclassifications may be inevitable. Because the number of subjects was limited, it was not possible to conduct examination-based analyses of site-specific cancer. Another limitation is the difficulties associated with applying the results of the present study directly to populations in other countries because this study was conducted solely on a Japanese population.

In conclusion, the present study suggests that the number of metabolic abnormalities is associated with the risk of cancer in obese Japanese adults. Moreover, hypertension and diabetes, but not dyslipidemia, may be key metabolic abnormalities contributing to the risk of cancer. The risk of each site-specific cancer may differ according to metabolic phenotypes. Further studies are warranted on the underlying mechanisms as well as the causal relationship between metabolic phenotypes and each site-specific cancer.

## Supplementary Material

Supplementary Data Supplementary Table S1-S14

Supplementary Data Supplementary File (List of the contributors to the J-MICC Study)
