# Supplementary material for: The Significance of Comprehensive Metabolic Phenotypes in Cancer Risk: A Japan Multi-Institutional Collaborative Cohort Study
Source: Cancer Res Commun. 2024 Nov 21;4(11):2986–97. doi: 10.1158/2767-9764.CRC-24-0249 (PMC11579844; doi:10.1158/2767-9764.CRC-24-0249)
Supplement: Supplementary Data — Supplementary Table S1-S14 [file crc-24-0249_supplementary_data_suppst1-st14.docx]

| **Supplementary Table 1. Background characteristics of participants according to sex and obesity classification.** | | | | | | | | | | | | | | |  |
| --- | --- | --- | --- | --- | --- | --- | --- | --- | --- | --- | --- | --- | --- | --- | --- |
| **Subjects for the analysis of Examination-based Metabolic phenotypes** | | | | | | |  | **Subjects for the analysis of Questionnaire-based Metabolic phenotypes** | | | | | | |  |
| Characteristics^a^ | Male | | *P*-value^b^ | Female | | *P*-value^b^ |  | Characteristics^a^ | Male | | *P*-value^b^ | Female | | *P*-value^b^ |  |
|  | Normal weight  (BMI<25) | Obesity  (BMI≥25) |  | Normal weight (BMI<25) | Obesity  (BMI≥25) |  |  |  | Normal weight (BMI<25) | Obesity  (BMI≥25) |  | Normal weight (BMI<25) | Obesity  (BMI≥25) |  |  |
|  | (*n* = 8657) | (*n* = 3812) |  | (*n* = 10391) | (*n* = 2497) |  |  |  | (*n* = 16517) | (*n* = 6727) |  | (*n* = 24966) | (*n* = 4832) |  |  |
| Age (years) | 57 (47, 63) | 55 (46, 62) | <0.0001 | 55 (46, 62) | 57 (49, 63) | <0.0001 |  | Age (years) | 57 (48, 63) | 54 (46, 62) | <0.0001 | 54 (45, 61) | 56 (48, 63) | <0.0001 |  |
| Exercise during leisure time (MET-hours/week) | 6.8 (0.9, 20.4) | 6.0 (0.4, 17.9) | <0.0001 | 5.1 (0 17.9) | 5.1 (0, 15.8) | 0.052 |  | Exercise during leisure time (MET-hours/week) | 6.8 (1.3, 18.8) | 5.3 (0.4, 17.9) | <0.0001 | 5.1 (0.4, 17.9) | 4.3 (0, 15.6) | <0.0001 |  |
| Educational background (years) | | | |  | | | | Educational background (years) | | | |  | | | |
| ≤9 | 1063 (12.3) | 479 (12.6) | 0.728 | 1177 (11.3) | 523 (21.0) | <0.0001 |  | ≤9 | 1561 (9.5) | 675 (10.0) | 0.414 | 1783 (7.1) | 707 (14.6) | <0.0001 |  |
| 10–15 | 4448 (51.4) | 1988 (52.2) |  | 7763 (74.7) | 1774 (71.1) |  |  | 10–15 | 8025 (48.6) | 3291 (48.9) |  | 19330 (77.4) | 3701 (76.6) |  |  |
| ≥16 | 3087 (35.7) | 1320 (34.6) |  | 1392 (13.4) | 185 (7.4) |  |  | ≥16 | 6843 (41.4) | 2728 (40.6) |  | 3739 (15.0) | 398 (8.2) |  |  |
| Unknown | 59 (0.7) | 25 (0.7) |  | 59 (0.6) | 15 (0.6) |  |  | Unknown | 88 (0.5) | 33 (0.5) |  | 114 (0.5) | 26 (0.5) |  |  |
| Smoking habit | | | |  | | | | Smoking habit | | | |  | | | |
| Current | 2429 (28.1) | 998 (26.2) | 0.030 | 595 (5.7) | 127 (5.1) | <0.0001 |  | Current | 4972 (30.1) | 1990 (29.6) | 0.011 | 1823 (7.3) | 316 (6.5) | 0.007 |  |
| Past | 3478 (40.2) | 1620 (42.5) |  | 749 (7.2) | 139 (5.6) |  |  | Past | 6584 (39.9) | 2819 (41.9) |  | 1943 (7.8) | 328 (6.8) |  |  |
| Never | 2750 (31.8) | 1194 (31.3) |  | 9047 (87.1) | 2231 (89.4) |  |  | Never | 4961 (30.0) | 1918 (28.5) |  | 21200 (84.9) | 4188 (86.7) |  |  |
| Pack-years |  |  |  |  |  |  |  | Pack-years |  |  |  |  |  |  |  |
| 0 | 2750 (31.8) | 1194 (31.3) | <0.0001 | 9047 (87.1) | 2231 (89.4) | <0.0001 |  | 0 | 4961 (30.4) | 1918 (28.5) | <0.0001 | 21200 (84.9) | 4188 (86.7) | <0.0001 |  |
| >0 and <20 | 2227 (25.7) | 812 (21.3) |  | 949 (9.1) | 177 (7.1) |  |  | >0 and <20 | 4097 (24.8) | 1416 (21.1) |  | 2597 (10.4) | 409 (8.5) |  |  |
| ≥20 | 3379 (39.0) | 1668 (43.8) |  | 314 (3.0) | 76 (3.0) |  |  | ≥20 | 6979 (42.3) | 3180 (47.3) |  | 984 (3.9) | 208 (4.3) |  |  |
| Unknown | 301 (3.5) | 138 (3.6) |  | 81 (0.8) | 13 (0.5) |  |  | Unknown | 480 (2.9) | 213 (3.2) |  | 185 (0.7) | 27 (0.6) |  |  |
| Alcohol drinking | | | |  | | | | Alcohol drinking | | | |  | | | |
| Never | 1779 (20.6) | 804 (21.1) | 0.002 | 6161 (59.3) | 1629 (65.2) | <0.0001 |  | Never | 3301 (20.0) | 1424 (21.2) | 0.0003 | 13960 (55.9) | 3083 (63.8) | <0.0001 |  |
| Past | 172 (2.0) | 74 (1.9) |  | 136 (1.3) | 32 (1.3) |  |  | Past | 432 (2.6) | 157 (2.3) |  | 416 (1.7) | 91 (1.9) |  |  |
| >0 and <20 g/day | 3161 (36.5) | 1261 (33.1) |  | 3424 (33.0) | 708 (28.4) |  |  | >0 and <20 g/day | 5830 (35.3) | 2192 (32.6) |  | 8853 (35.5) | 1408 (29.1) |  |  |
| ≥20 g/day | 3545 (41.0) | 1673 (43.9) |  | 670 (6.5) | 128 (5.1) |  |  | ≥20 g/day | 6954 (42.1) | 2954 (43.9) |  | 1737 (7.0) | 250 (5.2) |  |  |
| Menopausal status | | | |  |  |  |  | Menopausal status | | | |  |  |  |  |
| Premenopausal |  |  |  | 4083 (39.3) | 810 (32.4) | <0.0001 |  | Premenopausal |  |  |  | 10570 (42.3) | 1663 (34.4) | <0.0001 |  |
| Postmenopausal |  |  |  | 6250 (60.2) | 1669 (66.8) |  |  | Postmenopausal |  |  |  | 14302 (57.3) | 3147 (65.1) |  |  |
| Missing |  |  |  | 58 (0.6) | 18 (0.7) |  |  | Missing |  |  |  | 94 (0.4) | 22 (0.5) |  |  |
| Medical history | | | |  | | | | Medical history | | | |  | | | |
| Gastric ulcer | 1408 (16.3) | 527 (13.8) | 0.0005 | 905 (8.7) | 167 (6.7) | 0.001 |  | Gastric ulcer | 3062 (18.5) | 1088 (16.2) | <0.0001 | 2472 (9.9) | 397 (8.2) | 0.0003 |  |
| Chronic gastritis | 1073 (12.4) | 339 (8.9) | <0.0001 | 1174 (11.3) | 193 (7.7) | <0.0001 |  | Chronic gastritis | 2210 (13.4) | 737 (11.0) | <0.0001 | 3091 (12.4) | 442 (9.2) | <0.0001 |  |
| Colorectal polyps | 1070 (12.4) | 475 (12.5) | 0.875 | 578 (5.6) | 148 (5.9) | 0.478 |  | Colorectal polyps | 2070 (12.5) | 866 (12.9) | 0.478 | 1270 (5.1) | 269 (5.6) | 0.168 |  |
| Hepatitis B | 120 (1.4) | 71 (1.9) | 0.046 | 110 (1.1) | 21 (0.8) | 0.330 |  | Hepatitis B | 239 (1.5) | 122 (1.8) | 0.040 | 257 (1.0) | 56 (1.2) | 0.419 |  |
| Hepatitis C | 79 (0.9) | 34 (0.9) | 0.911 | 77 (0.7) | 19 (0.8) | 0.917 |  | Hepatitis C | 202 (1.2) | 85 (1.3) | 0.799 | 240 (1.0) | 58 (1.2) | 0.126 |  |
| Fatty liver | 740 (8.6) | 808 (21.2) | <0.0001 | 419 (4.0) | 364 (14.6) | <0.0001 |  | Fatty liver | 1618 (9.8) | 1590 (23.6) | <0.0001 | 921 (3.7) | 719 (14.9) | <0.0001 |  |
| Asthma | 481 (5.6) | 212 (5.6) | 0.991 | 675 (6.5) | 186 (7.5) | 0.087 |  | Asthma | 916 (5.6) | 395 (5.9) | 0.329 | 1684 (6.8) | 377 (7.8) | 0.008 |  |
| High blood pressure | 1591 (18.4) | 1174 (30.8) | <0.0001 | 1325 (12.8) | 734 (29.4) | <0.0001 |  | High blood pressure | 3047 (18.5) | 2096 (31.2) | <0.0001 | 2813 (11.3) | 1361 (28.2) | <0.0001 |  |
| Diabetes | 592 (6.8) | 370 (9.7) | <0.0001 | 243 (2.3) | 174 (7.0) | <0.0001 |  | Diabetes | 1211 (7.3) | 646 (9.6) | <0.0001 | 549 (2.2) | 324 (6.7) | <0.0001 |  |
| Dyslipidemia | 1153 (13.3) | 714 (18.7) | <0.0001 | 1468 (14.1) | 515 (20.6) | <0.0001 |  | Dyslipidemia | 2447 (14.8) | 1409 (21.0) | <0.0001 | 3472 (13.9) | 1034 (21.4) | <0.0001 |  |
| Medication | | | |  | | | | Medication | | | |  | | | |
| High blood pressure | 1289 (14.9) | 1016 (26.7) | <0.0001 | 1100 (10.6) | 667 (26.7) | <0.0001 |  | High blood pressure | 2447 (14.8) | 1771 (26.3) | <0.0001 | 2234 (9.0) | 1203 (24.9) | <0.0001 |  |
| Diabetes | 355 (4.1) | 263 (6.9) | <0.0001 | 149 (1.4) | 130 (5.2) | <0.0001 |  | Diabetes | 735 (4.5) | 470 (7.0) | <0.0001 | 336 (1.4) | 243 (5.0) | <0.0001 |  |
| High blood cholesterol | 570 (6.6) | 393 (10.3) | <0.0001 | 957 (9.2) | 400 (16.0) | <0.0001 |  | High blood cholesterol | 1051 (6.4) | 715 (10.6) | <0.0001 | 2064 (8.3) | 743 (15.4) | <0.0001 |  |
| Sleeping pills | 216 (2.5) | 106 (2.8) | 0.354 | 445 (4.3) | 115 (4.6) | 0.477 |  | Sleeping pills | 517 (3.1) | 201 (3.0) | 0.570 | 1179 (4.7) | 230 (4.8) | 0.911 |  |
| Antipyretic | 141 (1.6) | 55 (1.4) | 0.442 | 407 (3.9) | 142 (5.7) | <0.0001 |  | Antipyretic | 283 (1.7) | 132 (2.0) | 0.194 | 999 (4.0) | 263 (5.4) | <0.0001 |  |
| Laxative | 106 (1.2) | 56 (1.5) | 0.267 | 660 (6.4) | 137 (5.5) | 0.107 |  | Laxative | 272 (1.7) | 86 (1.3) | 0.039 | 1652 (6.6) | 291 (6.0) | 0.125 |  |
| MET, metabolic equivalent. |  |  |  |  |  |  |  |  |  |  |  |  |  |  |  |
| ^a^ Median (25%, 75%) or number of subjects (%). |  |  |  |  |  |  |  |  |  |  |  |  |  |  |  |
| ^b^ Wilcoxon’s rank sum test or Chi-square test. | |  |  |  |  |  |  |  | |  |  |  |  |  |  |

| **Supplementary Table 2. Multivariable hazard ratios and 95% confidence intervals for the association between metabolic syndrome, its components and cancer incidence.** | | | | | | | | |
| --- | --- | --- | --- | --- | --- | --- | --- | --- |
| **Examination-based** |  |  |  |  |  |  |  |  |
|  | Presence | Subjects | Incident  cancer cases | Person-years | Incidence rate  person/1000 person-years | HR^a^ (95% CI) | HR^b^ (95% CI) | HR^c^ (95% CI) |
| Metabolic syndrome | No | 21159 | 1226 | 163195.4 | 7.51 | 1 | 1 | 1 |
|  | Yes | 4198 | 358 | 33136.6 | 10.80 | **1.21 (1.07-1.36)** | **1.18 (1.04-1.33)** | **1.17 (1.04-1.33)** |
| Number of components | 0 | 7915 | 360 | 61627.2 | 5.84 | 1 | 1 | 1 |
|  | 1 | 7601 | 464 | 58163.6 | 7.98 | 1.03 (0.90-1.19) | 1.02 (0.88-1.17) | 1.02 (0.88-1.17) |
|  | 2 | 5643 | 402 | 43404.6 | 9.26 | 1.08 (0.93-1.25) | 1.05 (0.91-1.22) | 1.05 (0.91-1.22) |
|  | ≥3 | 4198 | 358 | 33136.6 | 10.80 | **1.25 (1.08-1.46)** | **1.20 (1.03-1.41)** | **1.20 (1.03-1.41)** |
|  |  |  |  |  |  | ***P*-trend =0.004** | ***P*-trend =0.017** | ***P*-trend =0.016** |
| Obesity | No | 19048 | 1135 | 145979.1 | 7.78 | 1 | 1 | 1 |
|  | Yes | 6309 | 449 | 50352.9 | 8.92 | **1.12 (1.00-1.25)** | 1.11 (0.99-1.24) | 1.11 (0.99-1.24) |
| High blood pressure | No | 13569 | 690 | 107426.3 | 6.42 | 1 | 1 | 1 |
|  | Yes | 11788 | 894 | 88905.7 | 10.06 | **1.11 (1.00-1.24)** | 1.10 (0.99-1.23) | 1.10 (0.99-1.22) |
| Elevated triglycerides | No | 20286 | 1238 | 157263.1 | 7.87 | 1 | 1 | 1 |
|  | Yes | 5071 | 346 | 39068.9 | 8.86 | 1.03 (0.92-1.17) | 1.00 (0.88-1.13) | 1.00 (0.88-1.13) |
| Low HDL-cholesterol | No | 23188 | 1437 | 179294.3 | 8.01 | 1 | 1 | 1 |
|  | Yes | 2169 | 147 | 17037.6 | 8.63 | 1.08 (0.91-1.29) | 1.09 (0.92-1.30) | 1.09 (0.92-1.30) |
| Elevated blood glucose | No | 17793 | 959 | 135977.9 | 7.05 | 1 | 1 | 1 |
|  | Yes | 7564 | 625 | 60354.0 | 10.36 | **1.14 (1.02-1.26)** | 1.11 (1.00-1.23) | 1.11 (1.00-1.23) |
| **Questionnaire-based** |  |  |  |  |  |  |  |  |
|  | Presence | Subjects | Incident  cancer cases | Person-years | Incidence rate  person/1000 person-years | HR^a^ (95% CI) | HR^b^ (95% CI) | HR^c^ (95% CI) |
| Number of components | 0 | 36476 | 2680 | 299603.5 | 8.95 | 1 | 1 | 1 |
|  | 1 | 11997 | 1231 | 97932.0 | 12.57 | 1.04 (0.97-1.12) | 1.04 (0.97-1.11) | 1.04 (0.97-1.17) |
|  | ≥2 | 4569 | 556 | 35944.3 | 15.47 | **1.15 (1.04-1.26)** | **1.13 (1.03-1.25)** | **1.13 (1.03-1.24)** |
|  |  |  |  |  |  | ***P*-trend =0.006** | ***P*-trend =0.011** | ***P*-trend =0.012** |
| Obesity | No | 41483 | 3394 | 337736.4 | 10.05 | 1 | 1 | 1 |
|  | Yes | 11559 | 1073 | 95743.4 | 11.21 | **1.10 (1.02-1.18)** | **1.09 (1.01-1.17)** | **1.09 (1.02-1.17)** |
| High blood pressure | No | 43417 | 3316 | 356768.7 | 9.29 | 1 | 1 | 1 |
|  | Yes | 9625 | 1151 | 76711.1 | 15.00 | **1.16 (1.08-1.24)** | **1.14 (1.06-1.22)** | **1.14 (1.06-1.22)** |
| Dyslipidemia | No | 43739 | 3603 | 358094.1 | 10.06 | 1 | 1 | 1 |
|  | Yes | 9303 | 864 | 75385.8 | 11.46 | 0.92 (0.85-0.99) | 0.93 (0.86-1.00) | 0.92 (0.86-1.00) |
| Diabetes | No | 50217 | 4063 | 411018.3 | 9.89 | 1 | 1 | 1 |
|  | Yes | 2825 | 404 | 22461.5 | 17.99 | **1.27 (1.15-1.41)** | **1.25 (1.12-1.39)** | **1.25 (1.13-1.39)** |
| HR, hazard ratio; CI, confidence interval; HDL, high-density lipoprotein. | | | | | | | | |
| ^a^ Adjusted for age, sex, research sites, and educational background. | | | |  |  |  |  |  |
| ^b^ Additionally adjusted for pack-years (four categories), drinking habit (four categories), and physical activity level (quartiles). | | | | | | |  |  |
| ^c^ Additionally adjusted for miso soup (quartiles), fruits (quartiles), and vegetables (quartiles) consumption. | | | | | |  |  |  |

| **Supplementary Table 3. Multivariable hazard ratios and 95% confidence intervals for the association between metabolic phenotypes and cancer incidence.** | | | | | |
| --- | --- | --- | --- | --- | --- |
| **Examination-based Metabolic phenotypes** | | | |  |  |
| **Normal Weight** |  |  |  |  |  |
|  | MHNW | MUNW  (No. of components=1) | MUNW  (No.of components=2) | MUNW  (No. of components≥3) | *P*-trend |
| Participants | 7915 | 6693 | 3464 | 976 |  |
| Parson-years | 61627.2 | 50718.5 | 26094.6 | 7538.7 |  |
| No. of cases | 360 | 426 | 266 | 83 |  |
| Model 1 HR^a^ (95 % CI) | 1.00 | 1.04 (0.90-1.20) | 1.08 (0.91-1.27) | 1.12 (0.88-1.43) | 0.277 |
| Model 2 HR^b^ (95 % CI) | 1.00 | 1.02 (0.88-1.17)  ) | 1.04 (0.88-1.23) | 1.05 (0.82-1.35) | 0.601 |
| Model 3 HR^c^ (95 % CI) | 1.00 | 1.02 (0.88-1.18) | 1.04 (0.88-1.23) | 1.06 (0.83-1.35) | 0.566 |
|  |  |  |  |  |  |
| **Obesity** |  |  |  |  |  |
|  | MHO |  | MUHO  (No. of components=obesity + 1) | MUHO  (No.of components≥ obesity+2) | *P*-trend |
| Participants | 908 |  | 2179 | 3222 |  |
| Parson-years | 7445.1 |  | 17310.0 | 25597.9 |  |
| No. of cases | 38 |  | 136 | 275 |  |
| Model 1 HR^a^ (95 % CI) | 1.00 |  | 1.18 (0.82-1.70) | **1.47 (1.04-2.09)** | **0.006** |
| Model 2 HR^b^ (95% CI) | 1.00 |  | 1.19 (0.83-1.71) | **1.46 (1.03-2.07)** | **0.009** |
| Model 3 HR^c^ (95 % CI) | 1.00 |  | 1.21 (0.84-1.74) | **1.47 (1.04-2.09)** | **0.009** |
|  |  |  |  |  |  |
| **Questionnaire-based Metabolic phenotypes** | |  |  |  |  |
| **Normal Weight** |  |  |  |  |  |
|  | MHNW | MUNW  (No. of components=1) | MUNW  (No.of components=2) | MUNW  (No. of components=3) | *P*-trend |
| Participants | 30099 | 8645 | 2444 | 295 |  |
| Parson-years | 246204.1 | 70002.5 | 19270.5 | 2259.3 |  |
| No. of cases | 2183 | 883 | 298 | 30 |  |
| Model 1 HR^a^ (95 % CI) | 1.00 | 1.03 (0.95-1.12) | 1.12 (0.99-1.26) | 0.88 (0.62-1.27) | 0.206 |
| Model 2 HR^b^ (95 % CI) | 1.00 | 1.03 (0.95-1.12) | 1.10 (0.97-1.24) | 0.86 (0.60-1.23) | 0.313 |
| Model 3 HR^c^ (95 % CI) | 1.00 | 1.03 (0.95-1.12  ) | 1.10 (0.97-1.24  ) | 0.86 (0.60-1.23) | 0.318 |
|  |  |  |  |  |  |
| **Obesity** |  |  |  |  |  |
|  | MHO |  | MUHO  (No. of components=obesity + 1) | MUHO  (No.of components≥ obesity+2) | *P*-trend |
| Participants | 6377 |  | 3352 | 1830 |  |
| Parson-years | 53399.4 |  | 27929.5 | 14414.5 |  |
| No. of cases | 497 |  | 348 | 228 |  |
| Model 1 HR^a^ (95 % CI) | 1.00 |  | 1.05 (0.92-1.21) | **1.19 (1.01-1.39)** | **0.047** |
| Model 2 HR^b^ (95% CI) | 1.00 |  | 1.05 (0.92-1.21) | **1.19 (1.01-1.40)** | **0.042** |
| Model 3 HR^c^ (95 % CI) | 1.00 |  | 1.05 (0.92-1.21) | **1.19 (1.01-1.40)** | **0.041** |
| HR, hazard ratio; CI, confidence interval; MHNW, Metabolically healthy normal weight; MUNW, Metabolically unhealthy normal weight; MHO Metabolically healthy obesity; MUHO, Metabolically unhealthy obesity. | | | | |  |
| ^a^ Adjusted for age, sex, research sites, and educational background. | | |  |  |  |
| ^b^ Additionally adjusted for pack-years (four categories), drinking habit (four categories), and physical activity level (quartiles). | | | |  |  |
| ^c^ Additionally adjusted for miso soup (quartiles), fruits (quartiles), and vegetables (quartiles) consumption. | | | |  |  |

| **Supplementary Table 4. Multivariable hazard ratios and 95% confidence intervals for the association between metabolic phenotypes and cancer incidence stratified by sex.** | | | | | |
| --- | --- | --- | --- | --- | --- |
| **Male** |  |  |  |  |  |
| **Examination-based Metabolic phenotypes** | | | | | |
| **Normal Weight** | | | | | |
|  | MHNW | MUNW (No. of components=1) | MUNW (No.of components=2) | MUNW (No. of components≥3) | *P*-trend |
| Participants | 2676 | 3228 | 2133 | 620 |  |
| Parson-years | 21355.6 | 25383.1 | 16440.9 | 4926.6 |  |
| No. of cases | 161 | 251 | 188 | 60 |  |
| Model 1 HR^a^ (95 % CI) | 1.00 | 0.98 (0.80-1.19) | 1.00 (0.81-1.24) | 1.04 (0.77-1.40) | 0.828 |
| Model 2 HR^b^ (95 % CI) | 1.00 | 0.94 (0.77-1.15) | 0.95 (0.77-1.18) | 0.96 (0.71-1.29) | 0.728 |
| Model 3 HR^c^ (95 % CI) | 1.00 | 0.95 (0.78-1.16) | 0.96 (0.77-1.18) | 0.96 (0.71-1.30) | 0.739 |
| **Obesity** | | | | | |
|  | MHO |  | MUHO (No. of components=obesity + 1) | MUHO (No.of components≥ obesity+2) | *P*-trend |
| Participants | 401 |  | 1201 | 2210 |  |
| Parson-years | 3316.2 |  | 9572.7 | 17641.7 |  |
| No. of cases | 11 |  | 83 | 197 |  |
| Model 1 HR^a^ (95 % CI) | 1.00 |  | **1.94 (1.03-3.64)** | **2.30 (1.25-4.24)** | **0.005** |
| Model 2 HR^b^ (95% CI) | 1.00 |  | **1.94 (1.03-3.65)** | **2.26 (1.22-4.16)** | **0.008** |
| Model 3 HR^c^ (95 % CI) | 1.00 |  | **2.00 (1.06-3.77)** | **2.32 (1.26-4.28)** | **0.006** |
|  |  |  |  |  |  |
| **Questionnaire-based Metabolic phenotypes** | | | | | |
| **Normal Weight** | | | | | |
|  | MHNW | MUNW (No. of components=1) | MUNW (No.of components=2) | MUNW (No. of components=3) | *P*-trend |
| Participants | 11083 | 4007 | 1237 | 190 |  |
| Parson-years | 89738.8 | 31636.5 | 9573.4 | 1441.0 |  |
| No. of cases | 1018 | 526 | 194 | 19 |  |
| Model 1 HR^a^ (95 % CI) | 1.00 | 1.05 (0.94-1.16) | 1.12 (0.96-1.31) | 0.70 (0.44-1.10) | 0.511 |
| Model 2 HR^b^ (95 % CI) | 1.00 | 1.04 (0.93-1.15) | 1.07 (0.91-1.25) | 0.66 (0.42-1.03) | 0.974 |
| Model 3 HR^c^ (95 % CI) | 1.00 | 1.04 (0.93-1.16) | 1.06 (0.91-1.24) | 0.66 (0.42-1.04) | 1.000 |
|  |  |  |  |  |  |
| **Obesity** | | | | | |
|  | MHO |  | MUHO (No. of components=obesity + 1) | MUHO (No.of components≥ obesity+2) | *P*-trend |
| Participants | 3597 |  | 2048 | 1082 |  |
| Parson-years | 29773.1 |  | 16816.2 | 8301.5 |  |
| No. of cases | 276 |  | 235 | 155 |  |
| Model 1 HR^a^ (95 % CI) | 1.00 |  | 1.14 (0.96-1.36) | **1.37 (1.12-1.68)** | **0.002** |
| Model 2 HR^b^ (95% CI) | 1.00 |  | 1.14 (0.95-1.36) | **1.37 (1.12-1.68)** | **0.003** |
| Model 3 HR^c^ (95 % CI) | 1.00 |  | 1.14 (0.95-1.36) | **1.37 (1.12-1.67)** | **0.003** |
|  |  |  |  |  |  |
|  | | | | | |
|  | | | | | |
|  | | | | | |
|  | | | | | |
|  | | | | | |
|  | | | | | |
|  | | | | | |
|  | | | | | |
|  | | | | | |
|  | | | | | |
|  | | | | | |
|  | | | | | |
|  | | | | | |
|  | | | | | |
|  | | | | | |
| **Female** | | | | | |
| **Examination-based Metabolic phenotypes** | | | | | |
| **Normal Weight** | | | | | |
|  | MHNW | MUNW (No. of components=1) | MUNW (No.of components=2) | MUNW (No. of components≥3) | *P*-trend |
| Participants | 5239 | 3465 | 1331 | 356 |  |
| Parson-years | 40271.6 | 25335.5 | 9653.7 | 2612.2 |  |
| No. of cases | 199 | 175 | 78 | 23 |  |
| Model 1 HR^a^ (95 % CI) | 1.00 | 1.20 (0.97-1.49) | 1.31 (1.00-1.73) | 1.39 (0.89-2.17) | **0.025** |
| Model 2 HR^b^ (95 % CI) | 1.00 | 1.19 (0.96-1.47) | 1.28 (0.97-1.69) | 1.35 (0.87-2.11) | **0.042** |
| Model 3 HR^c^ (95 % CI) | 1.00 | 1.19 (0.97-1.48) | 1.29 (0.98-1.70) | 1.37 (0.88-2.13) | **0.036** |
|  |  |  |  |  |  |
| **Obesity** | | | | | |
|  | MHO |  | MUHO (No. of components=obesity + 1) | MUHO (No.of components≥ obesity+2) | *P*-trend |
| Participants | 507 |  | 978 | 1012 |  |
| Parson-years | 4128.8 |  | 7737.3 | 7956.2 |  |
| No. of cases | 27 |  | 53 | 78 |  |
| Model 1 HR^a^ (95 % CI) | 1.00 |  | 0.90 (0.56-1.44) | 1.21 (0.77-1.90) | 0.218 |
| Model 2 HR^b^ (95% CI) | 1.00 |  | 0.91 (0.57-1.46) | 1.22 (0.78-1.92) | 0.202 |
| Model 3 HR^c^ (95 % CI) | 1.00 |  | 0.93 (0.58-1.50) | 1.25 (0.79-1.96) | 0.186 |
|  |  |  |  |  |  |
| **Questionnaire-based Metabolic phenotypes** | | | | | |
| **Normal Weight** | | | | | |
|  | MHNW | MUNW (No. of components=1) | MUNW (No.of components=2) | MUNW (No. of components=3) | *P*-trend |
| Participants | 19016 | 4638 | 1207 | 105 |  |
| Parson-years | 156465.3 | 38366.0 | 9697.0 | 818.4 |  |
| No. of cases | 1165 | 357 | 104 | 11 |  |
| Model 1 HR^a^ (95 % CI) | 1.00 | 1.07 (0.94-1.21) | 1.16 (0.94-1.43) | 1.41 (0.78-2.56) | 0.070 |
| Model 2 HR^b^ (95 % CI) | 1.00 | 1.07 (0.95-1.22) | 1.17 (0.95-1.44) | 1.45 (0.80-2.63) | 0.055 |
| Model 3 HR^c^ (95 % CI) | 1.00 | 1.07 (0.95-1.21) | 1.17 (0.95-1.44) | 1.42 (0.78-2.58) | 0.060 |
|  |  |  |  |  |  |
| **Obesity** | | | | | |
|  | MHO |  | MUHO (No. of components=obesity + 1) | MUHO (No.of components≥ obesity+2) | *P*-trend |
| Participants | 2780 |  | 1304 | 748 |  |
| Parson-years | 23626.2 |  | 11113.3 | 6113.0 |  |
| No. of cases | 221 |  | 113 | 73 |  |
| Model 1 HR^a^ (95 % CI) | 1.00 |  | 0.96 (0.76-1.21) | 1.00 (0.76-1.32) | 0.924 |
| Model 2 HR^b^ (95% CI) | 1.00 |  | 0.97 (0.77-1.23) | 1.01 (0.77-1.34) | 1.000 |
| Model 3 HR^c^ (95 % CI) | 1.00 |  | 0.97 (0.76-1.22) | 1.02 (0.77-1.35) | 0.956 |
| HR, hazard ratio; CI, confidence interval; MHNW, Metabolically healthy normal weight; MUNW, Metabolically unhealthy normal weight; MHO Metabolically healthy obesity;  MUHO, Metabolically unhealthy obesity. | | | | | |
| ^a^ Adjusted for age, research sites, and educational background. | | |  |  |  |
| ^b^ Additionally adjusted for pack-years (four categories), drinking habit (four categories), and physical activity level (quartiles). | | | |  |  |
| ^c^ Additionally adjusted for miso soup (quartiles), fruits (quartiles), and vegetables (quartiles) consumption. | | | |  |  |

| **Supplementary Table 5. Multivariable hazard ratios and 95% confidence intervals for the association between MetS components and cancer incidence stratified by obesity.** | | | | | | | | |
| --- | --- | --- | --- | --- | --- | --- | --- | --- |
|  |  |  |  |  |  |  |  |  |
| **Examination-based** | | | | | | | | |
| **Normal Weight** | | | | | | | | |
|  | **High blood pressure** | | **Elevated triglycerides** | | **Low HDL-cholesterol** | | **Elevated blood glucose** | |
|  | (-) | (+) | (-) | (+) | (-) | (+) | (-) | (+) |
| Participants | 11333 | 7715 | 16113 | 2935 | 17822 | 1226 | 14246 | 4802 |
| Parson-years | 88893.5 | 57085.6 | 123728.3 | 22250.8 | 136537.1 | 9441.9 | 108238.1 | 37741.0 |
| No. of cases | 565 | 570 | 939 | 196 | 1054 | 81 | 762 | 373 |
| Model 1 HR^a^ (95 % CI) | 1.00 | 1.10 (0.97-1.24) | 1.00 | 0.99 (0.85-1.16) | 1.00 | 1.04 (0.83-1.31) | 1.00 | 1.04 (0.92-1.18) |
| Model 2 HR^b^ (95% CI) | 1.00 | 1.09 (0.96-1.23) | 1.00 | 0.95 (0.81-1.11) | 1.00 | 1.05 (0.83-1.32) | 1.00 | 1.01 (0.89-1.15) |
| Model 3 HR^c^ (95% CI) | 1.00 | 1.09 (0.96-1.23) | 1.00 | 0.95 (0.81-1.11) | 1.00 | 1.05 (0.84-1.32) | 1.00 | 1.02 (0.89-1.16) |
|  |  |  |  |  |  |  |  |  |
| **Obesity** | | | | | | | | |
|  | **High blood pressure** | | **Elevated triglycerides** | | **Low HDL-cholesterol** | | **Elevated blood glucose** | |
|  | (-) | (+) | (-) | (+) | (-) | (+) | (-) | (+) |
| Participants | 2236 | 4073 | 4173 | 2136 | 5366 | 943 | 3547 | 2762 |
| Parson-years | 18532.8 | 31820.1 | 33534.8 | 16818.1 | 42757.2 | 7595.7 | 27739.8 | 22613.1 |
| No. of cases | 125 | 324 | 299 | 150 | 383 | 66 | 197 | 252 |
| Model 1 HR^a^ (95 % CI) | 1.00 | 1.08 (0.87-1.34) | 1.00 | 1.07 (0.87-1.31) | 1.00 | 1.10 (0.84-1.43) | 1.00 | **1.32 (1.09-1.60)** |
| Model 2 HR^b^ (95% CI) | 1.00 | 1.08 (0.87-1.34) | 1.00 | 1.06 (0.86-1.29) | 1.00 | 1.10 (0.84-1.44) | 1.00 | **1.30 (1.07-1.58)** |
| Model 3 HR^c^ (95% CI) | 1.00 | 1.07 (0.86-1.33) | 1.00 | 1.06 (0.87-1.30) | 1.00 | 1.09 (0.83-1.43) | 1.00 | **1.30 (1.07-1.58)** |
|  |  |  |  |  |  |  |  |  |
| **Questionnaire-based** | | | | | | | | |
| **Normal Weight** | | | | | | | | |
|  | **High blood pressure** | | **Dyslipidemia** | | **Diabetes** | |  |  |
|  | (-) | (+) | (-) | (+) | (-) | (+) |  |  |
| Participants | 35422 | 6061 | 34933 | 6550 | 39676 | 1807 |  |  |
| Parson-years | 289905.9 | 47830.5 | 284500.1 | 53236.3 | 323481.8 | 14254.6 |  |  |
| No. of cases | 2680 | 714 | 2802 | 592 | 3131 | 263 |  |  |
| Model 1 HR^a^ (95 % CI) | 1.00 | **1.12 (1.03-1.22)** | 1.00 | 0.90 (0.82-0.98) | 1.00 | **1.23 (1.09-1.40)** |  |  |
| Model 2 HR^b^ (95% CI) | 1.00 | **1.10 (1.01-1.19)** | 1.00 | 0.91 (0.83-0.99) | 1.00 | **1.20 (1.05-1.36)** |  |  |
| Model 3 HR^c^ (95% CI) | 1.00 | **1.09 (1.00-1.19)** | 1.00 | 0.91 (0.83-0.99) | 1.00 | **1.20 (1.06-1.37)** |  |  |
|  |  |  |  |  |  |  |  |  |
| **Obesity** | | | | | | | | |
|  | **High blood pressure** | | **Dyslipidemia** | | **Diabetes** | |  |  |
|  | (-) | (+) | (-) | (+) | (-) | (+) |  |  |
| Participants | 7995 | 3564 | 8806 | 2753 | 10541 | 1018 |  |  |
| Parson-years | 66862.7 | 28880.7 | 73594.0 | 22149.4 | 87536.5 | 8206.9 |  |  |
| No. of cases | 636 | 437 | 801 | 272 | 932 | 141 |  |  |
| Model 1 HR^a^ (95 % CI) | 1.00 | **1.19 (1.05-1.35)** | 1.00 | 0.94 (0.81-1.08) | 1.00 | **1.32 (1.10 - 1.58)** |  |  |
| Model 2 HR^b^ (95% CI) | 1.00 | **1.19 (1.05-1.35)** | 1.00 | 0.94 (0.82-1.09) | 1.00 | **1.33 (1.11 - 1.59)** |  |  |
| Model 3 HR^c^ (95% CI) | 1.00 | **1.19 (1.05-1.35)** | 1.00 | 0.94 (0.82-1.08) | 1.00 | **1.33 (1.11 - 1.59)** |  |  |
| HR, hazard ratio; CI, confidence interval. | |  |  |  |  |  |  |  |
| ^a^ Adjusted for age, sex, research sites and educational background. | | | |  |  |  |  |  |
| ^b^ Additionally adjusted for pack-years (four categories), drinking habit (four categories), and physical activity level (quartiles). | | | | | | |  |  |
| ^c^ Additionally adjusted for miso soup (quartiles), fruits (quartiles), and vegetables (quartiles) consumption. | | | | | |  |  |  |

| **Supplementary Table 6. Multivariable hazard ratios and 95% confidence intervals for the association between MetS components and cancer incidence stratified by obesity and sex.** | | | | | | | | |
| --- | --- | --- | --- | --- | --- | --- | --- | --- |
| **Male Subjects** |  |  |  |  |  |  |  |  |
| **Examination-based** |  |  |  |  |  |  |  |  |
| **Normal Weight** |  |  |  |  |  |  |  |  |
|  | **High blood pressure** | | **Elevated triglycerides** | | **Low HDL-cholesterol** | | **Elevated blood glucose** | |
|  | (-) | (+) | (-) | (+) | (-) | (+) | (-) | (+) |
| Participants | 4690 | 3967 | 6769 | 1888 | 8183 | 474 | 5574 | 3083 |
| Parson-years | 37951.9 | 30154.2 | 53338.6 | 14767.6 | 64283.9 | 3822.2 | 43339.1 | 24767.0 |
| No. of cases | 303 | 357 | 520 | 140 | 621 | 39 | 382 | 278 |
| Model 1 HR^a^ (95 % CI) | 1.00 | 1.02 (0.87-1.19) | 1.00 | 1.00 (0.83-1.20) | 1.00 | 1.05 (0.76-1.45) | 1.00 | 1.02 (0.87-1.19) |
| Model 2 HR^b^ (95% CI) | 1.00 | 1.00 (0.85-1.17) | 1.00 | 0.95 (0.78-1.14) | 1.00 | 1.06 (0.76-1.47) | 1.00 | 0.99 (0.85-1.16) |
| Model 3 HR^c^ (95% CI) | 1.00 | 1.00 (0.85-1.17) | 1.00 | 0.95 (0.78-1.14) | 1.00 | 1.06 (0.76-1.47) | 1.00 | 1.00 (0.85-1.17) |
|  |  |  |  |  |  |  |  |  |
| **Obesity** |  |  |  |  |  |  |  |  |
|  | **High blood pressure** | | **Elevated triglycerides** | | **Low HDL-cholesterol** | | **Elevated blood glucose** | |
|  | (-) | (+) | (-) | (+) | (-) | (+) | (-) | (+) |
| Participants | 1273 | 2539 | 2226 | 1586 | 3373 | 439 | 1886 | 1926 |
| Parson-years | 10624.0 | 19906.6 | 17933.7 | 12596.9 | 27042.7 | 3487.9 | 14687.1 | 15843.5 |
| No. of cases | 68 | 223 | 173 | 118 | 257 | 34 | 112 | 179 |
| Model 1 HR^a^ (95 % CI) | 1.00 | 1.21 (0.91-1.60) | 1.00 | 1.17 (0.92-1.48) | 1.00 | 1.18 (0.83-1.70) | 1.00 | 1.23 (0.97-1.57) |
| Model 2 HR^b^ (95% CI) | 1.00 | 1.20 (0.90-1.60) | 1.00 | 1.15 (0.91-1.47) | 1.00 | 1.18 (0.82-1.71) | 1.00 | 1.21 (0.95-1.54) |
| Model 3 HR^c^ (95% CI) | 1.00 | 1.19 (0.90-1.59) | 1.00 | 1.16 (0.91-1.48) | 1.00 | 1.17 (0.81-1.69) | 1.00 | 1.22 (0.96-1.56) |
|  |  |  |  |  |  |  |  |  |
| **Questionnaire-based** |  |  |  |  |  |  |  |  |
| **Normal Weight** |  |  |  |  |  |  |  |  |
|  | **High blood pressure** | | **Dyslipidemia** |  | **Diabetes** |  |  |  |
|  | (-) | (+) | (-) | (+) | (-) | (+) |  |  |
| Participants | 13349 | 3168 | 13876 | 2641 | 15275 | 1242 |  |  |
| Parson-years | 108101.3 | 24288.5 | 113008.4 | 21465.2 | 122813.4 | 9985.9 |  |  |
| No. of cases | 1297 | 460 | 1463 | 294 | 1540 | 217 |  |  |
| Model 1 HR^a^ (95 % CI) | 1.00 | 1.06 (0.95-1.18) | 1.00 | **0.88 (0.78-1.00)** | 1.00 | 1.22 (1.05-1.40) |  |  |
| Model 2 HR^b^ (95% CI) | 1.00 | 1.01 (0.91-1.13) | 1.00 | 0.89 (0.78-1.00) | 1.00 | 1.18 (1.02-1.36) |  |  |
| Model 3 HR^c^ (95% CI) | 1.00 | 1.01 (0.91-1.13) | 1.00 | 0.88 (0.78-1.00) | 1.00 | 1.17 (1.01-1.35) |  |  |
|  |  |  |  |  |  |  |  |  |
| **Obesity** |  |  |  |  |  |  |  |  |
|  | **High blood pressure** | | **Dyslipidemia** |  | **Diabetes** |  |  |  |
|  | (-) | (+) | (-) | (+) | (-) | (+) |  |  |
| Participants | 4562 | 2165 | 5160 | 1567 | 10541 | 1018 |  |  |
| Parson-years | 37732.5 | 25107.5 | 42509.0 | 12381.8 | 49482.7 | 5408.2 |  |  |
| No. of cases | 366 | 300 | 489 | 177 | 565 | 101 |  |  |
| Model 1 HR^a^ (95 % CI) | 1.00 | **1.23 (1.05-1.44)** | 1.00 | 1.14 (0.96-1.35) | 1.00 | **1.32 (1.07 - 1.63)** |  |  |
| Model 2 HR^b^ (95% CI) | 1.00 | **1.21 (1.04-1.42)** | 1.00 | 1.14 (0.96-1.36) | 1.00 | **1.33 (1.07 - 1.64)** |  |  |
| Model 3 HR^c^ (95% CI) | 1.00 | **1.21 (1.03-1.42)** | 1.00 | 1.14 (0.96-1.37) | 1.00 | **1.32 (1.07 - 1.64)** |  |  |
|  |  |  |  |  |  |  |  |  |
|  |  |  |  |  |  |  |  |  |
|  |  |  |  |  |  |  |  |  |
|  |  |  |  |  |  |  |  |  |
|  |  |  |  |  |  |  |  |  |
|  |  |  |  |  |  |  |  |  |
|  |  |  |  |  |  |  |  |  |
|  |  |  |  |  |  |  |  |  |
| **Female subjects** |  |  |  |  |  |  |  |  |
| **Examination-based** |  |  |  |  |  |  |  |  |
| **Normal Weight** |  |  |  |  |  |  |  |  |
|  | **High blood pressure** | | **Elevated triglycerides** | | **Low HDL-cholesterol** | | **Elevated blood glucose** | |
|  | (-) | (+) | (-) | (+) | (-) | (+) | (-) | (+) |
| Participants | 6643 | 3748 | 9344 | 1047 | 9639 | 752 | 8672 | 1719 |
| Parson-years | 50941.5 | 26931.4 | 70389.7 | 7483.2 | 72253.2 | 5619.7 | 64899.0 | 12974.0 |
| No. of cases | 262 | 213 | 419 | 56 | 433 | 42 | 380 | 95 |
| Model 1 HR^a^ (95 % CI) | 1.00 | **1.28 (1.06-1.56)** | 1.00 | 1.10 (0.83-1.46) | 1.00 | 1.16 (0.84-1.60) | 1.00 | 1.09 (0.87-1.37) |
| Model 2 HR^b^ (95% CI) | 1.00 | **1.28 (1.05-1.56)** | 1.00 | 1.07 (0.80-1.42) | 1.00 | 1.14 (0.82-1.57) | 1.00 | 1.07 (0.85-1.35) |
| Model 3 HR^c^ (95% CI) | 1.00 | **1.29 (1.06-1.56)** | 1.00 | 1.07 (0.80-1.42) | 1.00 | 1.14 (0.83-1.58) | 1.00 | 1.08 (0.86-1.36) |
|  |  |  |  |  |  |  |  |  |
| **Obesity** |  |  |  |  |  |  |  |  |
|  | **High blood pressure** | | **Elevated triglycerides** | | **Low HDL-cholesterol** | | **Elevated blood glucose** | |
|  | (-) | (+) | (-) | (+) | (-) | (+) | (-) | (+) |
| Participants | 963 | 1534 | 1947 | 550 | 1993 | 504 | 1663 | 836 |
| Parson-years | 7908.9 | 11913.5 | 15601.1 | 4221.3 | 15714.5 | 4107.9 | 13052.8 | 6769.6 |
| No. of cases | 57 | 101 | 126 | 32 | 126 | 32 | 85 | 73 |
| Model 1 HR^a^ (95 % CI) | 1.00 | 0.95 (0.67-1.33) | 1.00 | 0.89 (0.60-1.31) | 1.00 | 1.04 (0.70-1.54) | 1.00 | **1.52 (1.10-2.08)** |
| Model 2 HR^b^ (95% CI) | 1.00 | 0.95 (0.68-1.34) | 1.00 | 0.89 (0.60-1.32) | 1.00 | 1.04 (0.70-1.55) | 1.00 | **1.53 (1.11-2.11)** |
| Model 3 HR^c^ (95% CI) | 1.00 | 0.97 (0.69-1.36) | 1.00 | 0.89 (0.60-1.33) | 1.00 | 1.04 (0.70-1.55) | 1.00 | **1.54 (1.12-2.11)**  **-2.11)** |
|  |  |  |  |  |  |  |  |  |
| **Questionnaire-based** |  |  |  |  |  |  |  |  |
| **Normal Weight** |  |  |  |  |  |  |  |  |
|  | **High blood pressure** | | **Dyslipidemia** | | **Diabetes** | |  |  |
|  | (-) | (+) | (-) | (+) | (-) | (+) |  |  |
| Participants | 22073 | 6061 | 21057 | 3909 | 24401 | 565 |  |  |
| Parson-years | 181804.7 | 23542.0 | 173351.7 | 31994.9 | 200668.5 | 4678.2 |  |  |
| No. of cases | 1383 | 254 | 1339 | 298 | 1591 | 46 |  |  |
| Model 1 HR^a^ (95 % CI) | 1.00 | **1.22 (1.07-1.41)** | 1.00 | 1.01 (0.88-1.15) | 1.00 | 1.07 (0.80-1.44) |  |  |
| Model 2 HR^b^ (95% CI) | 1.00 | **1.23 (1.07-1.41)** | 1.00 | 1.02 (0.89-1.16) | 1.00 | 1.07 (0.80-1.44) |  |  |
| Model 3 HR^c^ (95% CI) | 1.00 | **1.22 (1.06-1.41)** | 1.00 | 1.02 (0.89-1.16) | 1.00 | 1.07 (0.80-1.44) |  |  |
|  |  |  |  |  |  |  |  |  |
| **Obesity** |  |  |  |  |  |  |  |  |
|  | **High blood pressure** | | **Dyslipidemia** | | **Diabetes** | |  |  |
|  | (-) | (+) | (-) | (+) | (-) | (+) |  |  |
| Participants | 3433 | 1399 | 3646 | 1186 | 4497 | 335 |  |  |
| Parson-years | 29130.3 | 11722.3 | 31085.0 | 9767.6 | 38053.8 | 2798.8 |  |  |
| No. of cases | 270 | 137 | 312 | 95 | 367 | 40 |  |  |
| Model 1 HR^a^ (95 % CI) | 1.00 | 1.15 (0.93-1.42) | 1.00 | **0.75 (0.59-0.96)** | 1.00 | 1.35 (0.97 - 1.88) |  |  |
| Model 2 HR^b^ (95% CI) | 1.00 | 1.16 (0.93-1.44) | 1.00 | **0.76 (0.60-0.97)** | 1.00 | 1.35 (0.97 - 1.87) |  |  |
| Model 3 HR^c^ (95% CI) | 1.00 | 1.16 (0.94-1.44) | 1.00 | **0.76 (0.60-0.97)** | 1.00 | 1.36 (0.98 - 1.90) |  |  |
| HR, hazard ratio; CI, confidence interval. | |  |  |  |  |  |  |  |
| ^a^ Adjusted for age, research sites, and educational background. | |  |  |  |  |  |  |  |
| ^b^ Additionally adjusted for pack-years (four categories), drinking habit (four categories), and physical activity level (quartiles). | | | | | | |  |  |
| ^c^ Additionally adjusted for miso soup (quartiles), fruits (quartiles), and vegetables (quartiles) consumption. | | | | | |  |  |  |

| **Supplementary Table 7. Multivariable hazard ratios and 95% confidence intervals for the association between metabolic phenotypes and cancer incidence handling subjects who had cancer within one or two years as censored.** | | | | | |
| --- | --- | --- | --- | --- | --- |
|  |  |  |  |  |  |
| **Examination-based Metabolic phenotypes (one year)** | | | | | |
| **Normal Weight** | | | | | |
|  | MHNW | MUNW (No. of components=1) | MUNW (No.of components=2) | MUNW (No. of components≥3) | *P*-trend |
| Participants | 7915 | 6693 | 3464 | 976 |  |
| Parson-years | 61627.2 | 50718.5 | 26094.6 | 7538.7 |  |
| No. of cases | 329 | 385 | 238 | 76 |  |
| Model 1 HR^a^ (95 % CI) | 1.00 | 1.02 (0.88-1.19) | 1.05 (0.88-1.25) | 1.11 (0.86-1.44) | 0.399 |
| Model 2 HR^b^ (95 % CI) | 1.00 | 1.00 (0.86-1.17) | 1.01 (0.85-1.21) | 1.05 (0.81-1.36) | 0.752 |
| Model 3 HR^c^ (95 % CI) | 1.00 | 1.01 (0.86-1.17) | 1.02 (0.85-1.21) | 1.05 (0.81-1.36) | 0.718 |
|  |  |  |  |  |  |
| **Obesity** | | | | | |
|  | MHO |  | MUHO (No. of components=obesity + 1) | MUHO (No.of components≥ obesity+2) | *P*-trend |
| Participants | 908 |  | 2179 | 3222 |  |
| Parson-years | 7445.1 |  | 17310.0 | 25597.9 |  |
| No. of cases | 34 |  | 118 | 251 |  |
| Model 1 HR^a^ (95 % CI) | 1.00 |  | 1.14 (0.78-1.68) | **1.49 (1.04-2.16)** | **0.004** |
| Model 2 HR^b^ (95% CI) | 1.00 |  | 1.15 (0.78-1.69) | **1.48 (1.03-2.14)** | **0.006** |
| Model 3 HR^c^ (95 % CI) | 1.00 |  | 1.16 (0.79-1.71) | **1.48 (1.03-2.14)** | **0.007** |
|  |  |  |  |  |  |
| **Questionnaire-based Metabolic phenotypes (one year)** | | | | | |
| **Normal Weight** | | | | | |
|  | MHNW | MUNW (No. of components=1) | MUNW (No.of components=2) | MUNW (No. of components=3) | *P*-trend |
| Participants | 30099 | 8645 | 2444 | 295 |  |
| Parson-years | 246204.1 | 70002.5 | 19270.5 | 2259.3 |  |
| No. of cases | 1534 | 657 | 221 | 22 |  |
| Model 1 HR^a^ (95 % CI) | 1.00 | 1.07 (0.97-1.17) | 1.15 (0.99-1.33) | 0.89 (0.59-1.36) | 0.094 |
| Model 2 HR^b^ (95 % CI) | 1.00 | 1.07 (0.97-1.17) | 1.13 (0.98-1.30) | 0.87 (0.57-1.32) | 0.144 |
| Model 3 HR^c^ (95 % CI) | 1.00 | 1.07 (0.97-1.17) | 1.13 (0.98-1.30) | 0.87 (0.57-1.33) | 0.145 |
|  |  |  |  |  |  |
| **Obesity** | | | | | |
|  | MHO |  | MUHO (No. of components=obesity + 1) | MUHO (No.of components≥ obesity+2) | *P*-trend |
| Participants | 6377 |  | 3352 | 1830 |  |
| Parson-years | 53399.4 |  | 27929.5 | 14414.5 |  |
| No. of cases | 364 |  | 272 | 169 |  |
| Model 1 HR^a^ (95 % CI) | 1.00 |  | 1.10 (0.94-1.29) | 1.20 (0.99-1.45) | 0.052 |
| Model 2 HR^b^ (95% CI) | 1.00 |  | 1.10 (0.94-1.29) | **1.21 (1.00-1.46)** | **0.044** |
| Model 3 HR^c^ (95 % CI) | 1.00 |  | 1.10 (0.94-1.29) | **1.21 (1.01-1.47)** | **0.042** |
|  |  |  |  |  |  |
|  | | | | | |
|  | | | | | |
|  | | | | | |
|  | | | | | |
|  | | | | | |
|  | | | | | |
|  | | | | | |
|  | | | | | |
|  | | | | | |
|  | | | | | |
|  | | | | | |
|  | | | | | |
|  | | | | | |
|  | | | | | |
| **Examination-based Metabolic phenotypes (two years)** | | | | | |
| **Normal Weight** | | | | | |
|  | MHNW | MUNW (No. of components=1) | MUNW (No.of components=2) | MUNW (No. of components≥3) | *P*-trend |
| Participants | 7915 | 6693 | 3464 | 976 |  |
| Parson-years | 61627.2 | 50718.5 | 26094.6 | 7538.7 |  |
| No. of cases | 290 | 341 | 211 | 72 |  |
| Model 1 HR^a^ (95 % CI) | 1.00 | 1.04 (0.88-1.22) | 1.06 (0.88-1.28) | 1.20 (0.92-1.57) | 0.217 |
| Model 2 HR^b^ (95 % CI) | 1.00 | 1.02 (0.86-1.19) | 1.03 (0.85-1.24) | 1.14 (0.87-1.48) | 0.439 |
| Model 3 HR^c^ (95 % CI) | 1.00 | 1.02 (0.87-1.20) | 1.03 (0.86-1.25) | 1.14 (0.88-1.49) | 0.406 |
|  |  |  |  |  |  |
| **Obesity** | | | | | |
|  | MHO |  | MUHO (No. of components=obesity + 1) | MUHO (No.of components≥ obesity+2) | *P*-trend |
| Participants | 908 |  | 2179 | 3222 |  |
| Parson-years | 7445.1 |  | 17310.0 | 25597.9 |  |
| No. of cases | 29 |  | 110 | 227 |  |
| Model 1 HR^a^ (95 % CI) | 1.00 |  | 1.23 (0.82-1.86) | **1.55 (1.04-2.30)** | 0.007 |
| Model 2 HR^b^ (95% CI) | 1.00 |  | 1.25 (0.83-1.89) | **1.54 (1.03-2.28)** | **0.011** |
| Model 3 HR^c^ (95 % CI) | 1.00 |  | 1.26 (0.84-1.91) | **1.54 (1.03-2.28)** | **0.013** |
|  |  |  |  |  |  |
| **Questionnaire-based Metabolic phenotypes (two years)** | | | | | |
| **Normal Weight** | | | | | |
|  | MHNW | MUNW (No. of components=1) | MUNW (No.of components=2) | MUNW (No. of components=3) | *P*-trend |
| Participants | 30099 | 8645 | 2444 | 295 |  |
| Parson-years | 246204.1 | 70002.5 | 19270.5 | 2259.3 |  |
| No. of cases | 1357 | 581 | 205 | 17 |  |
| Model 1 HR^a^ (95 % CI) | 1.00 | 1.08 (0.97-1.19) | **1.22 (1.05-1.42)** | 0.79 (0.49-1.28) | **0.044** |
| Model 2 HR^b^ (95 % CI) | 1.00 | 1.08 (0.98-1.19) | **1.20 (1.03-1.39)** | 0.77 (0.48-1.25) | 0.064 |
| Model 3 HR^c^ (95 % CI) | 1.00 | 1.08 (0.97-1.19) | **1.20 (1.03-1.39)** | 0.77 (0.48-1.25) | 0.065 |
|  |  |  |  |  |  |
| **Obesity** | | | | | |
|  | MHO |  | MUHO (No. of components=obesity + 1) | MUHO (No.of components≥ obesity+2) | *P*-trend |
| Participants | 6377 |  | 3352 | 1830 |  |
| Parson-years | 53399.4 |  | 27929.5 | 14414.5 |  |
| No. of cases | 328 |  | 239 | 155 |  |
| Model 1 HR^a^ (95 % CI) | 1.00 |  | 1.07 (0.90-1.27) | **1.22 (1.01-1.49)** | 0.052 |
| Model 2 HR^b^ (95% CI) | 1.00 |  | 1.07 (0.90-1.27) | **1.24 (1.02-1.51)** | **0.040** |
| Model 3 HR^c^ (95 % CI) | 1.00 |  | 1.07 (0.90-1.27) | **1.24 (1.02-1.51)** | **0.040** |
| HR, hazard ratio; CI, confidence interval; MHNW, Metabolically healthy normal weight; MUNW, Metabolically unhealthy normal weight; MHO Metabolically healthy obesity;  MUHO, Metabolically unhealthy obesity. | | | | |  |
| ^a^ Adjusted for age, sex, research sites, and educational background. | | |  |  |  |
| ^b^ Additionally adjusted for pack-years (four categories), drinking habit (four categories), and physical activity level (quartiles). | | | |  |  |
| ^c^ Additionally adjusted for miso soup (quartiles), fruits (quartiles), and vegetables (quartiles) consumption. | | | | | |

| **Supplementary Table 8. Multivariable hazard ratios and 95% confidence intervals for the association between MetS components and cancer incidence stratified by obesity handling subjects who had cancer within one year or two years as censored.** | | | | | | | | |
| --- | --- | --- | --- | --- | --- | --- | --- | --- |
|  |  |  |  |  |  |  |  |  |
| **Examination-based　(one year)** | | | | | | | | |
| **Normal Weight** | | | | | | | | |
|  | **High blood pressure** | | **Elevated triglycerides** | | **Low HDL-cholesterol** |  | **Elevated blood glucose** |  |
|  | (-) | (+) | (-) | (+) | (-) | (+) | (-) | (+) |
| Participants | 11333 | 7715 | 16113 | 2935 | 17822 | 1226 | 14246 | 4802 |
| Parson-years | 88893.5 | 57085.6 | 123728.3 | 22250.8 | 136537.1 | 9441.9 | 108238.1 | 37741.0 |
| No. of cases | 517 | 511 | 851 | 177 | 952 | 76 | 692 | 336 |
| Model 1 HR^a^ (95 % CI) | 1.00 | 1.08 (0.95-1.23) | 1.00 | 0.99 (0.84-1.17) | 1.00 | 1.08 (0.86-1.37) | 1.00 | 1.02 (0.89-1.17) |
| Model 2 HR^b^ (95% CI) | 1.00 | 1.07 (0.94-1.22) | 1.00 | 0.94 (0.80-1.11) | 1.00 | 1.08 (0.85-1.37) | 1.00 | 1.00 (0.87-1.14) |
| Model 3 HR^c^ (95% CI) | 1.00 | 1.07 (0.94-1.22) | 1.00 | 0.95 (0.80-1.12) | 1.00 | 1.08 (0.85-1.37) | 1.00 | 1.00 (0.87-1.14) |
|  |  |  |  |  |  |  |  |  |
| **Obesity** | | | | | | | | |
|  | **High blood pressure** | | **Elevated triglycerides** | | **Low HDL-cholesterol** |  | **Elevated blood glucose** |  |
|  | (-) | (+) | (-) | (+) | (-) | (+) | (-) | (+) |
| Participants | 2236 | 4073 | 4173 | 2136 | 5366 | 943 | 3547 | 2762 |
| Parson-years | 18532.8 | 31820.1 | 33534.8 | 16818.1 | 42757.2 | 7595.7 | 27739.8 | 22613.1 |
| No. of cases | 110 | 293 | 266 | 137 | 344 | 59 | 176 | 227 |
| Model 1 HR^a^ (95 % CI) | 1.00 | 1.11 (0.88-1.39) | 1.00 | 1.11 (0.90-1.37) | 1.00 | 1.09 (0.82-1.44) | 1.00 | **1.31 (1.07-1.61)** |
| Model 2 HR^b^ (95% CI) | 1.00 | 1.10 (0.87-1.39) | 1.00 | 1.10 (0.89-1.36) | 1.00 | 1.10 (0.83-1.46) | 1.00 | **1.30 (1.06-1.59)** |
| Model 3 HR^c^ (95% CI) | 1.00 | 1.09 (0.87-1.38) | 1.00 | 1.10 (0.89-1.37) | 1.00 | 1.08 (0.81-1.43) | 1.00 | **1.29 (1.06-1.58)** |
|  |  |  |  |  |  |  |  |  |
| **Questionnaire-based (one year)** | | | | | | |  |  |
| **Normal Weight** | | | | | | |  |  |
|  | **High blood pressure** | | **Dyslipidemia** |  | **Diabetes** |  |  |  |
|  | (-) | (+) | (-) | (+) | (-) | (+) |  |  |
| Participants | 35422 | 6061 | 34933 | 6550 | 39676 | 1807 |  |  |
| Parson-years | 289905.9 | 47830.5 | 284500.1 | 53236.3 | 323481.8 | 14254.6 |  |  |
| No. of cases | 1911 | 523 | 1989 | 445 | 2237 | 197 |  |  |
| Model 1 HR^a^ (95 % CI) | 1.00 | **1.13 (1.02-1.25)** | 1.00 | 0.92 (0.83-1.02) | 1.00 | **1.28 (1.11-1.49)** |  |  |
| Model 2 HR^b^ (95% CI) | 1.00 | **1.11 (1.01-1.23)** | 1.00 | 0.93 (0.84-1.03) | 1.00 | **1.24 (1.07-1.44)** |  |  |
| Model 3 HR^c^ (95% CI) | 1.00 | **1.11 (1.01-1.23)** | 1.00 | 0.93 (0.84-1.03) | 1.00 | **1.24 (1.07-1.44)** |  |  |
|  |  |  |  |  |  |  |  |  |
| **Obesity** | | | | | | |  |  |
|  | **High blood pressure** | | **Dyslipidemia** |  | **Diabetes** |  |  |  |
|  | (-) | (+) | (-) | (+) | (-) | (+) |  |  |
| Participants | 7995 | 3564 | 8806 | 2753 | 10541 | 1018 |  |  |
| Parson-years | 66862.7 | 28880.7 | 73594.0 | 22149.4 | 87536.5 | 8206.9 |  |  |
| No. of cases | 473 | 332 | 605 | 200 | 694 | 111 |  |  |
| Model 1 HR^a^ (95 % CI) | 1.00 | **1.19 (1.03-1.37)** | 1.00 | 0.94 (0.80-1.11) | 1.00 | **1.37 (1.12-1.67)** |  |  |
| Model 2 HR^b^ (95% CI) | 1.00 | **1.19 (1.03-1.37)** | 1.00 | 0.95 (0.81-1.12) | 1.00 | **1.37 (1.12-1.68)** |  |  |
| Model 3 HR^c^ (95% CI) | 1.00 | **1.19 (1.03-1.38)** | 1.00 | 0.95 ( 0.81-1.12) | 1.00 | **1.38 (1.12-1.69)** |  |  |
|  |  |  |  |  |  |  |  |  |
|  |  |  |  |  |  |  |  |  |
|  |  |  |  |  |  |  |  |  |
|  |  |  |  |  |  |  |  |  |
|  |  |  |  |  |  |  |  |  |
|  |  |  |  |  |  |  |  |  |
|  |  |  |  |  |  |  |  |  |
|  |  |  |  |  |  |  |  |  |
|  |  |  |  |  |  |  |  |  |
|  |  |  |  |  |  |  |  |  |
|  |  |  |  |  |  |  |  |  |
|  |  |  |  |  |  |  |  |  |
| **Examination-based　(two years)** | | | | | | | |  |
| **Normal Weight** | | | | | | | |  |
|  | **High blood pressure** | | **Elevated triglycerides** | | **Low HDL-cholesterol** |  | **Elevated blood glucose** |  |
|  | (-) | (+) | (-) | (+) | (-) | (+) | (-) | (+) |
| Participants | 11333 | 7715 | 16113 | 2935 | 17822 | 1226 | 14246 | 4802 |
| Parson-years | 88893.5 | 57085.6 | 123728.3 | 22250.8 | 136537.1 | 9441.9 | 108238.1 | 37741.0 |
| No. of cases | 461 | 453 | 752 | 162 | 844 | 70 | 609 | 305 |
| Model 1 HR^a^ (95 % CI) | 1.00 | 1.09 (0.95-1.24) | 1.00 | 1.03 (0.87-1.22) | 1.00 | 1.13 (0.88-1.45) | 1.00 | 1.04 (0.90-1.20) |
| Model 2 HR^b^ (95% CI) | 1.00 | 1.08 (0.94-1.24) | 1.00 | 0.98 (0.83-1.17) | 1.00 | 1.13 (0.88-1.45) | 1.00 | 1.02 (0.88-1.18) |
| Model 3 HR^c^ (95% CI) | 1.00 | 1.08 (0.94-1.24) | 1.00 | 0.99 (0.83-1.17) | 1.00 | 1.13 (0.88-1.45) | 1.00 | 1.02 (0.89-1.18) |
|  |  |  |  |  |  |  |  |  |
| **Obesity** | | | | | | | | |
|  | **High blood pressure** | | **Elevated triglycerides** | | **Low HDL-cholesterol** |  | **Elevated blood glucose** |  |
|  | (-) | (+) | (-) | (+) | (-) | (+) | (-) | (+) |
| Participants | 2236 | 4073 | 4173 | 2136 | 5366 | 943 | 3547 | 2762 |
| Parson-years | 18532.8 | 31820.1 | 33534.8 | 16818.1 | 42757.2 | 7595.7 | 27739.8 | 22613.1 |
| No. of cases | 97 | 269 | 242 | 124 | 311 | 55 | 158 | 208 |
| Model 1 HR^a^ (95 % CI) | 1.00 | 1.15 (0.91-1.47) | 1.00 | 1.11 (0.89-1.39) | 1.00 | 1.14 (0.85-1.52) | 1.00 | **1.31 (1.06-1.61)** |
| Model 2 HR^b^ (95% CI) | 1.00 | 1.15 (0.90-1.46) | 1.00 | 1.10 (0.88-1.37) | 1.00 | 1.14 (0.85-1.53) | 1.00 | **1.29 (1.04-1.60)** |
| Model 3 HR^c^ (95% CI) | 1.00 | 1.14 (0.89-1.45) | 1.00 | 1.10 (0.88-1.37) | 1.00 | 1.11 (0.83-1.50) | 1.00 | **1.29 (1.04-1.59)** |
|  |  |  |  |  |  |  |  |  |
| **Questionnaire-based (two years)** | | | | | | |  |  |
| **Normal Weight** | | | | | | |  |  |
|  | **High blood pressure** | | **Dyslipidemia** |  | **Diabetes** |  |  |  |
|  | (-) | (+) | (-) | (+) | (-) | (+) |  |  |
| Participants | 35422 | 6061 | 34933 | 6550 | 39676 | 1807 |  |  |
| Parson-years | 289905.9 | 47830.5 | 284500.1 | 53236.3 | 323481.8 | 14254.6 |  |  |
| No. of cases | 1700 | 460 | 1760 | 400 | 1978 | 182 |  |  |
| Model 1 HR^a^ (95 % CI) | 1.00 | **1.13 (1.01-1.25)** | 1.00 | 0.94 (0.84-1.05) | 1.00 | **1.35 (1.16-1.58)** |  |  |
| Model 2 HR^b^ (95% CI) | 1.00 | **1.11 (1.00-1.24)** | 1.00 | 0.95 (0.85-1.06) | 1.00 | **1.31 (1.12-1.53)** |  |  |
| Model 3 HR^c^ (95% CI) | 1.00 | **1.11 (1.00-1.24)** | 1.00 | 0.95 (0.85-1.06) | 1.00 | **1.31 (1.12-1.53)** |  |  |
|  |  |  |  |  |  |  |  |  |
| **Obesity** |  |  |  |  |  |  |  |  |
|  | **High blood pressure** | | **Dyslipidemia** |  | **Diabetes** |  |  |  |
|  | (-) | (+) | (-) | (+) | (-) | (+) |  |  |
| Participants | 7995 | 3564 | 8806 | 2753 | 10541 | 1018 |  |  |
| Parson-years | 66862.7 | 28880.7 | 73594.0 | 22149.4 | 87536.5 | 8206.9 |  |  |
| No. of cases | 425 | 297 | 541 | 181 | 620 | 102 |  |  |
| Model 1 HR^a^ (95 % CI) | 1.00 | **1.18 (1.01-1.37)** | 1.00 | 0.96 (0.81-1.14) | 1.00 | **1.40 (1.14-1.73)** |  |  |
| Model 2 HR^b^ (95% CI) | 1.00 | **1.18 (1.01-1.37)** | 1.00 | 0.97 (0.82-1.16) | 1.00 | **1.41 (1.14-1.75)** |  |  |
| Model 3 HR^c^ (95% CI) | 1.00 | **1.18 (1.01-1.37)** | 1.00 | 0.97 ( 0.82-1.16) | 1.00 | **1.42 (1.15-1.75)** |  |  |
| HR, hazard ratio; CI, confidence interval. | | | | | | |  |  |
| ^a^ Adjusted for age, sex, research sites, and educational background. | | | | | | |  |  |
| ^b^ Additionally adjusted for pack-years (four categories), drinking habit (four categories), and physical activity level (quartiles). | | | | | | |  |  |
| ^c^ Additionally adjusted for miso soup (quartiles), fruits (quartiles), and vegetables (quartiles) consumption. | | | | | | |  |  |

| **Supplementary Table 9. Multivariable hazard ratios and 95% confidence intervals for the association between metabolic phenotypes and total and site-specific cancer.** | | | | | |  |
| --- | --- | --- | --- | --- | --- | --- |
|  | **Examination-based analyses** | |  |  |  |  |
|  | **Total** |  |  |  |  |  |
|  |  | MHNW | MHO | MUNW | MUHO |  |
|  | Participants | 7915 | 908 | 11133 | 5401 |  |
|  | Parson-years | 61627.2 | 7445.1 | 84351.9 | 42907.8 |  |
|  | No. of cases | 360 | 38 | 775 | 411 |  |
|  | Model 1 HR^a^ (95 % CI) | 1.00 | 0.90(0.65-1.26) | 1.06 (0.93-1.21) | **1.20 (1.04-1.39)** |  |
|  | Model 2 HR^b^ (95 % CI) | 1.00 | 0.90 (0.64-1.25) | 1.04 (0.91-1.19) | **1.17 (1.01-1.36)** |  |
|  | Model 3 HR^c^ (95 % CI) | 1.00 | 0.90 (0.64-1.25) | 1.04 (0.91-1.19) | **1.17 (1.01-1.36)** |  |
|  |  |  |  |  |  |  |
|  | **Questionaire-based analyses** | |  |  |  |  |
|  | **Total** |  |  |  |  |  |
|  |  | MHNW | MHO | MUNW | MUHO |  |
|  | Participants | 30099 | 6377 | 11384 | 5182 |  |
|  | Parson-years | 246204.1 | 53399.4 | 91532.3 | 42344.0 |  |
|  | No. of cases | 2183 | 497 | 1211 | 576 |  |
|  | Model 1 HR^a^ (95 % CI) | 1.00 | 1.06 (0.96-1.17) | 1.05 (0.97-1.13) | **1.17 (1.06-1.28)** |  |
|  | Model 2 HR^b^ (95 % CI) | 1.00 | 1.06 (0.96-1.17) | 1.05 (0.97-1.12) | **1.15 (1.05-1.26)** |  |
|  | Model 3 HR^c^ (95 % CI) | 1.00 | 1.06 (0.96-1.17) | 1.05 (0.97-1.12) | **1.15 (1.04-1.26)** |  |
|  | **Stomach** |  |  |  |  |  |
|  |  | MHNW | MHO | MUNW | MUHO |  |
|  | Participants | 30099 | 6377 | 11384 | 5182 |  |
|  | Parson-years | 246204.1 | 53399.4 | 91532.3 | 42344.0 |  |
|  | No. of cases | 253 | 47 | 178 | 67 |  |
|  | Model 1 HR^a^ (95 % CI) | 1.00 | 0.78 (0.57-1.07) | 1.13 (0.93-1.37) | 0.95 (0.72-1.24) |  |
|  | Model 2 HR^b^ (95 % CI) | 1.00 | 0.77 (0.56-1.05) | 1.13 (0.92-1.37) | 0.93 (0.71-1.22) |  |
|  | Model 3 HR^c^ (95 % CI) | 1.00 | 0.76 (0.56-1.04) | 1.13 (0.92-1.37) | 0.93 (0.71-1.22) |  |
|  | **Colon and rectum** |  |  |  |  |  |
|  |  | MHNW | MHO | MUNW | MUHO |  |
|  | Participants | 30099 | 6377 | 11384 | 5182 |  |
|  | Parson-years | 246204.1 | 53399.4 | 91532.3 | 42344.0 |  |
|  | No. of cases | 258 | 79 | 159 | 91 |  |
|  | Model 1 HR^a^ (95 % CI) | 1.00 | **1.41 (1.10-1.82)** | 1.10 (0.90-1.35) | **1.48 (1.16-1.89)** |  |
|  | Model 2 HR^b^ (95 % CI) | 1.00 | **1.41 (1.09-1.82)** | 1.09 (0.89-1.34) | **1.45 (1.14-1.86)** |  |
|  | Model 3 HR^c^ (95 % CI) | 1.00 | **1.41 (1.09-1.82)** | 1.09 (0.89-1.34) | **1.45 (1.13-1.85)** |  |
|  | Model 4 HR^e^ (95 % CI) | 1.00 | **1.41 (1.09-1.82)** | 1.09 (0.89-1.34) | **1.44 (1.12-1.84)** |  |
|  | **Liver** |  |  |  |  |  |
|  |  | MHNW | MHO | MUNW | MUHO |  |
|  | Participants | 30099 | 6377 | 11384 | 5182 |  |
|  | Parson-years | 246204.1 | 53399.4 | 91532.3 | 42344.0 |  |
|  | No. of cases | 38 | 23 | 36 | 27 |  |
|  | Model 1 HR^a^ (95 % CI) | 1.00 | **2.40 (1.43-4.05)** | 1.57 (0.99-2.50) | **2.44 (1.47-4.03)** |  |
|  | Model 2 HR^b^ (95 % CI) | 1.00 | **2.38 (1.41-4.01)** | 1.54 (0.97-2.46) | **2.38 (1.44-3.95)** |  |
|  | Model 3 HR^c^ (95 % CI) | 1.00 | **2.39 (1.41-4.03)** | 1.54 (0.97-2.46) | **2.36 (1.43-3.92)** |  |
|  | Model 4 HR^f^ (95 % CI) | 1.00 | **2.12 (1.24-3.62)** | 1.43 (0.89-2.28) | **2.15 (1.30-3.57)** |  |
|  |  |  |  |  |  |  |
|  |  |  |  |  |  |  |
|  |  |  |  |  |  |  |
|  | **Pancreas** |  |  |  |  |  |
|  |  | MHNW | MHO | MUNW | MUHO |  |
|  | Participants | 30099 | 6377 | 11384 | 5182 |  |
|  | Parson-years | 246204.1 | 53399.4 | 91532.3 | 42344.0 |  |
|  | No. of cases | 64 | 14 | 63 | 24 |  |
|  | Model 1 HR^a^ (95 % CI) | 1.00 | 1.04 (0.58-1.87) | **1.58 (1.11-2.26)** | 1.50 (0.93-2.41) |  |
|  | Model 2 HR^b^ (95 % CI) | 1.00 | 1.04 (0.58-1.87) | **1.57 (1.10-2.25)** | 1.49 (0.92-2.40) |  |
|  | Model 3 HR^c^ (95 % CI) | 1.00 | 1.06 (0.59-1.89) | **1.60 (1.12-2.29)** | 1.51 (0.94-2.44) |  |
|  | **Lung and Bronchus** |  |  |  |  |  |
|  |  | MHNW | MHO | MUNW | MUHO |  |
|  | Participants | 30099 | 6377 | 11384 | 5182 |  |
|  | Parson-years | 246204.1 | 53399.4 | 91532.3 | 42344.0 |  |
|  | No. of cases | 234 | 59 | 137 | 56 |  |
|  | Model 1 HR^a^ (95 % CI) | 1.00 | 1.13 (0.85-1.51) | 0.95 (0.77-1.18) | 0.91 (0.68-1.22) |  |
|  | Model 2 HR^b^ (95 % CI) | 1.00 | 1.09 (0.82-1.45) | 0.94 (0.75-1.16) | 0.86 (0.64-1.15) |  |
|  | Model 3 HR^c^ (95 % CI) | 1.00 | 1.09 (0.82-1.46) | 0.94 (0.75-1.16) | 0.86 (0.64-1.16) |  |
|  |  |  |  |  |  |  |
|  | **Male** |  |  |  |  |  |
|  | **Total** |  |  |  |  |  |
|  |  | MHNW | MHO | MUNW | MUHO |  |
|  | Participants | 11083 | 3597 | 5434 | 3130 |  |
|  | Parson-years | 89738.8 | 29773.1 | 42650.9 | 25117.7 |  |
|  | No. of cases | 1018 | 276 | 739 | 390 |  |
|  | Model 1 HR^d^ (95 % CI) | 1.00 | 0.97 (0.85-1.11) | 1.06 (0.97-1.17) | **1.15 (1.02-1.29)** |  |
|  | Model 2 HR^b^ (95 % CI) | 1.00 | 0.95 (0.83-1.09) | 1.05 (0.95-1.15) | 1.11 (0.99-1.25) |  |
|  | Model 3 HR^c^ (95 % CI) | 1.00 | 0.95 (0.83-1.09) | 1.05 (0.95-1.15) | 1.11 (0.99-1.25) |  |
|  | **Prostate** |  |  |  |  |  |
|  |  | MHNW | MHO | MUNW | MUHO |  |
|  | Participants | 11083 | 3597 | 5434 | 3130 |  |
|  | Parson-years | 89738.8 | 29773.1 | 42650.9 | 25117.7 |  |
|  | No. of cases | 214 | 41 | 145 | 88 |  |
|  | Model 1 HR^d^ (95 % CI) | 1.00 | 0.74 (0.53-1.03) | 0.91 (0.74-1.13) | 1.21 (0.94-1.56) |  |
|  | Model 2 HR^b^ (95 % CI) | 1.00 | 0.76 (0.54-1.06) | 0.91 (0.73-1.12) | 1.23 (0.96-1.58) |  |
|  | Model 3 HR^c^ (95 % CI) | 1.00 | 0.75 (0.54-1.05) | 0.91 (0.74-1.13) | 1.22 (0.95-1.57) |  |
|  |  |  |  |  |  |  |
|  |  |  |  |  |  |  |
|  |  |  |  |  |  |  |
|  |  |  |  |  |  |  |
|  |  |  |  |  |  |  |
|  |  |  |  |  |  |  |
|  |  |  |  |  |  |  |
|  |  |  |  |  |  |  |
|  |  |  |  |  |  |  |
|  |  |  |  |  |  |  |
|  |  |  |  |  |  |  |
|  |  |  |  |  |  |  |
|  |  |  |  |  |  |  |
|  |  |  |  |  |  |  |
|  |  |  |  |  |  |  |
|  | **Female** |  |  |  |  |  |
|  | **Total** |  |  |  |  |  |
|  |  | MHNW | MHO | MUNW | MUHO |  |
|  | Participants | 19016 | 2780 | 5950 | 2052 |  |
|  | Parson-years | 156465.3 | 23626.2 | 48881.4 | 17226.3 |  |
|  | No. of cases | 1165 | 221 | 472 | 186 |  |
|  | Model 1 HR^d^ (95 % CI) | 1.00 | **1.37 (1.18-1.58)** | 1.09 (0.98-1.23) | **1.31 (1.12-1.54)** |  |
|  | Model 2 HR^b^ (95 % CI) | 1.00 | **1.37 (1.18-1.58)** | 1.10 (0.98-1.23) | **1.31 (1.12-1.54)** |  |
|  | Model 3 HR^c^ (95 % CI) | 1.00 | **1.37 (1.19-1.59)** | 1.10 (0.98-1.23) | **1.32 (1.12-1.55)** |  |
|  | **Breast** |  |  |  |  |  |
|  |  | MHNW | MHO | MUNW | MUHO |  |
|  | Participants | 19016 | 2780 | 5950 | 2052 |  |
|  | Parson-years | 156465.3 | 23626.2 | 48881.4 | 17226.3 |  |
|  | No. of cases | 370 | 71 | 121 | 59 |  |
|  | Model 1 HR^d^ (95 % CI) | 1.00 | **1.44 (1.11-1.86)** | 1.10 (0.89-1.38) | **1.61 (1.21-2.14)** |  |
|  | Model 2 HR^b^ (95 % CI) | 1.00 | **1.44 (1.11-1.86)** | 1.10 (0.89-1.37) | **1.62 (1.21-2.15)** |  |
|  | Model 3 HR^c^ (95 % CI) | 1.00 | **1.45 (1.12-1.88)** | 1.10 (0.89-1.38) | **1.63 (1.22-2.17)** |  |
|  | Model 4 HR^g^ (95 % CI) | 1.00 | **1.44 (1.12-1.87)** | 1.11 (0.89-1.38) | **1.61 (1.21-2.15)** |  |
|  | **Corpus uteri** |  |  |  |  |  |
|  |  | MHNW | MHO | MUNW | MUHO |  |
|  | Participants | 19016 | 2780 | 5950 | 2052 |  |
|  | Parson-years | 156465.3 | 23626.2 | 48881.4 | 17226.3 |  |
|  | No. of cases | 65 | 11 | 17 | 13 |  |
|  | Model 1 HR^d^ (95 % CI) | 1.00 | 1.33 (0.70-2.53) | 0.74 (0.42-1.30) | **1.90 (1.02-3.52)** |  |
|  | Model 2 HR^b^ (95 % CI) | 1.00 | 1.34 (0.70-2.55) | 0.75 (0.43-1.31) | **1.93 (1.04-3.58)** |  |
|  | Model 3 HR^c^ (95 % CI) | 1.00 | 1.36 (0.71-2.59) | 0.75 (0.43-1.31) | **1.95 (1.04-3.63)** |  |
|  | Model 4 HR^h^ (95 % CI) | 1.00 | 1.32 (0.69-2.51) | 0.76 (0.43-1.35) | **1.95 (1.04-3.66)** |  |
|  | HR, hazard ratio; CI, confidence interval; MHNW, Metabolically healthy normal weight; MUNW, Metabolically unhealthy normal weight; MHO Metabolically healthy obesity; MUHO, Metabolically unhealthy obesity. | | | | | |
|  | ^a^ Adjusted for age, sex, research sites, and educational background. | | | | | |
|  | ^b^ Additionally adjusted for pack-years (four categories), drinking habit (four categories), and physical activity level (quartiles). | | | | | |
|  | ^c^ Additionally adjusted for miso soup (quartiles), fruits (quartiles), and vegetables (quartiles) consumption. | | | | | |
|  | ^d^ Adjusted for age, research sites, and educational background. | | | | | |
|  | ^e^ Additionally adjusted for antipyretic use (two categories), calcium (quartiles), red meat (quartiles), and, processed meat (quartiles) consumption. | | | | | |
|  | ^f^ Additionally adjusted for history of hepatitis B (two categories) and hepatitis C (two categories). | | | | | |
|  | ^g^ Additionally adjusted for hormone replacement therapy (two categories), age of menarche (four categories), and age of menopause (five categories). | | | | | |
|  | ^h^ Additionally adjusted for hormone replacement therapy (two categories), age of menarche (four categories), age of menopause (five categories), and history of ovarian disease (three categories). | | | | | |

| **Supplementary Table 10. Multivariable hazard ratios and 95% confidence intervals for the association between components and pancreatic cancer incidence in normal weight subjects.** | | | | | | |
| --- | --- | --- | --- | --- | --- | --- |
| **Normal Weight** |  |  |  |  |  |  |
|  | MHNW | MUNW (No. of components=1) | MUNW (No.of components≥2) | *P*-trend |  |  |
| Participants | 30099 | 8645 | 2739 |  |  |  |
| Parson-years | 246204.1 | 70002.5 | 21529.8 |  |  |  |
| No. of cases | 64 | 43 | 20 |  |  |  |
| Model 1 HR^a^ (95 % CI) | 1.00 | 1.46 (0.99-2.17) | **1.86 (1.11-3.11)** | 0.011 |  |  |
| Model 2 HR^b^ (95 % CI) | 1.00 | 1.45 (0.98-2.16) | **1.80 (1.07-3.02)** | 0.015 |  |  |
| Model 3 HR^c^ (95 % CI) | 1.00 | 1.48 (0.99-2.20) | **1.82 (1.08-3.05)** | 0.013 |  |  |
|  |  |  |  |  |  |  |
| **Normal Weight** |  |  |  |  |  |  |
|  | **High blood pressure** |  | **Dyslipidemia** |  | **Diabetes** |  |
|  | (-) | (+) | (-) | (+) | (-) | (+) |
| Participants | 35422 | 6061 | 34933 | 6550 | 39676 | 1807 |
| Parson-years | 289905.9 | 47830.5 | 284500.1 | 53236.3 | 323481.8 | 14254.6 |
| No. of cases | 95 | 32 | 97 | 30 | 104 | 23 |
| Model 1 HR^a^ (95 % CI) | 1.00 | 1.15 (0.76-1.73) | 1.00 | 1.23 (0.81-1.87) | 1.00 | **2.59 (1.62-4.12)** |
| Model 2 HR^b^ (95% CI) | 1.00 | 1.13 (0.75-1.72) | 1.00 | 1.24 (0.82-1.88) | 1.00 | **2.42 (1.51-3.86)** |
| Model 3 HR^c^ (95% CI) | 1.00 | 1.15 (0.76-1.74) | 1.00 | 1.24 (0.82-1.89) | 1.00 | **2.43 (1.52-3.89)** |
| HR, hazard ratio; CI, confidence interval. | | | | | | |
| ^a^ Adjusted for age, sex, research sites and educational background. | | | | | | |
| ^b^ Additionally adjusted for pack-years (four categories), drinking habit (four categories), and physical activity level (quartiles). | | | | | | |
| ^c^ Additionally adjusted for miso soup (quartiles), fruits (quartiles), and vegetables (quartiles) consumption. | | | | | | |

| **Supplementary Table 11. Multivariable hazard ratios and 95% confidence intervals for the association between metabolic phenotypes and total and site-specific cancer handling subjects who had cancer within one year or two years as censored.** | | | | | | |
| --- | --- | --- | --- | --- | --- | --- |
|  | **Examination-based analyses** | |  |  |  |  |
|  | **Total (one year)** |  |  |  |  |  |
|  |  | MHNW | MHO | MUNW | MUHO |  |
|  | Participants | 7915 | 908 | 11133 | 5401 |  |
|  | Parson-years | 61627.2 | 7445.1 | 84351.9 | 42907.8 |  |
|  | No. of cases | 329 | 34 | 699 | 369 |  |
|  | Model 1 HR^a^ (95 % CI) | 1.00 | 0.87 (0.61-1.24) | 1.05 (0.91-1.20) | 1.16 (0.99-1.35) |  |
|  | Model 2 HR^b^ (95 % CI) | 1.00 | 0.87 (0.61-1.23) | 1.02 (0.89-1.17) | 1.13 (0.97-1.32) |  |
|  | Model 3 HR^c^ (95 % CI) | 1.00 | 0.86 (0.61-1.23) | 1.02 (0.89-1.17) | 1.13 (0.97-1.32) |  |
|  | **Total (two years)** |  |  |  |  |  |
|  |  | MHNW | MHO | MUNW | MUHO |  |
|  | Participants | 7915 | 908 | 11133 | 5401 |  |
|  | Parson-years | 61627.2 | 7445.1 | 84351.9 | 42907.8 |  |
|  | No. of cases | 290 | 29 | 624 | 337 |  |
|  | Model 1 HR^a^ (95 % CI) | 1.00 | 0.84 (0.57-1.23) | 1.06 (0.92-1.23) | **1.19 (1.01-1.40)** |  |
|  | Model 2 HR^b^ (95 % CI) | 1.00 | 0.83 (0.57-1.22) | 1.04 (0.90-1.20) | 1.16 (0.98-1.37) |  |
|  | Model 3 HR^c^ (95 % CI) | 1.00 | 0.83 (0.56-1.21) | 1.04 (0.90-1.20) | 1.16 (0.98-1.36) |  |
|  |  |  |  |  |  |  |
|  | **Questionaire-based analyses** | |  |  |  |  |
|  | **Total (one year)** |  |  |  |  |  |
|  |  | MHNW | MHO | MUNW | MUHO |  |
|  | Participants | 30099 | 6377 | 11384 | 5182 |  |
|  | Parson-years | 246204.1 | 53399.4 | 91532.3 | 42344.0 |  |
|  | No. of cases | 1534 | 364 | 900 | 441 |  |
|  | Model 1 HR^a^ (95 % CI) | 1.00 | 1.05 (0.94-1.18) | 1.08 (0.99-1.17) | **1.19 (1.07-1.33)** |  |
|  | Model 2 HR^b^ (95 % CI) | 1.00 | 1.04 (0.93-1.17) | 1.08 (0.99-1.17) | **1.18 (1.06-1.31)** |  |
|  | Model 3 HR^c^ (95 % CI) | 1.00 | 1.04 (0.93-1.17) | 1.08 (0.99-1.17) | **1.18 (1.06-1.31)** |  |
|  | **Total (two years)** |  |  |  |  |  |
|  |  | MHNW | MHO | MUNW | MUHO |  |
|  | Participants | 30099 | 6377 | 11384 | 5182 |  |
|  | Parson-years | 246204.1 | 53399.4 | 91532.3 | 42344.0 |  |
|  | No. of cases | 1357 | 328 | 803 | 394 |  |
|  | Model 1 HR^a^ (95 % CI) | 1.00 | 1.06 (0.94-1.20) | 1.09 (1.00-1.20) | **1.20 (1.07-1.35)** |  |
|  | Model 2 HR^b^ (95 % CI) | 1.00 | 1.05 (0.93-1.19) | 1.09 (1.00-1.20) | **1.19 (1.06-1.33)** |  |
|  | Model 3 HR^c^ (95 % CI) | 1.00 | 1.05 (0.93-1.19) | 1.09 (1.00-1.20) | **1.19 (1.06-1.33)** |  |
|  | **Stomach (one year)** |  |  |  |  |  |
|  |  | MHNW | MHO | MUNW | MUHO |  |
|  | Participants | 30099 | 6377 | 11384 | 5182 |  |
|  | Parson-years | 246204.1 | 53399.4 | 91532.3 | 42344.0 |  |
|  | No. of cases | 195 | 40 | 142 | 55 |  |
|  | Model 1 HR^a^ (95 % CI) | 1.00 | 0.84 (0.59-1.18) | 1.17 (0.94-1.46) | 0.98 (0.72-1.32) |  |
|  | Model 2 HR^b^ (95 % CI) | 1.00 | 0.82 (0.58-1.15) | 1.17 (0.94-1.46) | 0.96 (0.71-1.30) |  |
|  | Model 3 HR^c^ (95 % CI) | 1.00 | 0.82 (0.58-1.15) | 1.17 (0.94-1.46) | 0.96 (0.71-1.30) |  |
|  |  |  |  |  |  |  |
|  |  |  |  |  |  |  |
|  |  |  |  |  |  |  |
|  |  |  |  |  |  |  |
|  |  |  |  |  |  |  |
|  | **Stomach (two years)** |  |  |  |  |  |
|  |  | MHNW | MHO | MUNW | MUHO |  |
|  | Participants | 30099 | 6377 | 11384 | 5182 |  |
|  | Parson-years | 246204.1 | 53399.4 | 91532.3 | 42344.0 |  |
|  | No. of cases | 172 | 34 | 128 | 45 |  |
|  | Model 1 HR^a^ (95 % CI) | 1.00 | 0.80 (0.55-1.15) | 1.21 (0.96-1.53) | 0.91 (0.65-1.26) |  |
|  | Model 2 HR^b^ (95 % CI) | 1.00 | 0.78 (0.54-1.13) | 1.21 (0.96-1.53) | 0.89 (0.64-1.24) |  |
|  | Model 3 HR^c^ (95 % CI) | 1.00 | 0.78 (0.54-1.13) | 1.21 (0.96-1.53) | 0.89 (0.64-1.24) |  |
|  | **Colon and rectum (one year)** | |  |  |  |  |
|  |  | MHNW | MHO | MUNW | MUHO |  |
|  | Participants | 30099 | 6377 | 11384 | 5182 |  |
|  | Parson-years | 246204.1 | 53399.4 | 91532.3 | 42344.0 |  |
|  | No. of cases | 198 | 55 | 125 | 73 |  |
|  | Model 1 HR^a^ (95 % CI) | 1.00 | 1.25 (0.92-1.68) | 1.13 (0.90-1.42) | **1.51 (1.15-1.99)** |  |
|  | Model 2 HR^b^ (95 % CI) | 1.00 | 1.23 (0.91-1.67) | 1.12 (0.89-1.41) | **1.48 (1.13-1.95)** |  |
|  | Model 3 HR^c^ (95 % CI) | 1.00 | 1.23 (0.91-1.67) | 1.12 (0.89-1.41) | **1.48 (1.13-1.95)** |  |
|  | Model 4 HR^e^ (95 % CI) | 1.00 | 1.23 (0.91-1.67) | 1.12 (0.89-1.41) | **1.47 (1.12-1.94)** |  |
|  | **Colon and rectum (two years)** | |  |  |  |  |
|  |  | MHNW | MHO | MUNW | MUHO |  |
|  | Participants | 30099 | 6377 | 11384 | 5182 |  |
|  | Parson-years | 246204.1 | 53399.4 | 91532.3 | 42344.0 |  |
|  | No. of cases | 176 | 51 | 111 | 66 |  |
|  | Model 1 HR^a^ (95 % CI) | 1.00 | 1.30 (0.95-1.78) | 1.14 (0.89-1.46) | **1.55 (1.16-2.08)** |  |
|  | Model 2 HR^b^ (95 % CI) | 1.00 | 1.30 (0.95-1.78) | 1.14 (0.90-1.46) | **1.54 (1.15-2.05)** |  |
|  | Model 3 HR^c^ (95 % CI) | 1.00 | 1.29 (0.94-1.77) | 1.14 (0.90-1.46) | **1.54 (1.15-2.06)** |  |
|  | Model 4 HR^e^ (95 % CI) | 1.00 | 1.29 (0.94-1.77) | 1.14 (0.90-1.46) | **1.53 (1.14-2.04)** |  |
|  | **Liver (one year)** |  |  |  |  |  |
|  |  | MHNW | MHO | MUNW | MUHO |  |
|  | Participants | 30099 | 6377 | 11384 | 5182 |  |
|  | Parson-years | 246204.1 | 53399.4 | 91532.3 | 42344.0 |  |
|  | No. of cases | 28 | 17 | 27 | 23 |  |
|  | Model 1 HR^a^ (95 % CI) | 1.00 | **2.39 (1.30-4.40)** | 1.54 (0.90-2.64) | **2.70 (1.54-4.73)** |  |
|  | Model 2 HR^b^ (95 % CI) | 1.00 | **2.32 (1.26-4.27)** | 1.52 (0.89-2.60) | **2.59 (1.48-4.56)** |  |
|  | Model 3 HR^c^ (95 % CI) | 1.00 | **2.33 (1.27-4.28)** | 1.52 (0.89-2.61) | **2.54 (1.44-4.46)** |  |
|  | Model 4 HR^f^ (95 % CI) | 1.00 | **2.19 (1.18-4.06)** | 1.43 (0.83-2.46) | **2.28 (1.29-4.02)** |  |
|  | **Liver (two years)** |  |  |  |  |  |
|  |  | MHNW | MHO | MUNW | MUHO |  |
|  | Participants | 30099 | 6377 | 11384 | 5182 |  |
|  | Parson-years | 246204.1 | 53399.4 | 91532.3 | 42344.0 |  |
|  | No. of cases | 26 | 14 | 25 | 23 |  |
|  | Model 1 HR^a^ (95 % CI) | 1.00 | **2.12 (1.10-4.08)** | 1.55 (0.89-2.70) | **2.93 (1.65-5.19)** |  |
|  | Model 2 HR^b^ (95 % CI) | 1.00 | **2.05 (1.06-3.95)** | 1.53 (0.88-2.68) | **2.81 (1.59-4.99)** |  |
|  | Model 3 HR^c^ (95 % CI) | 1.00 | **2.05 (1.06-3.95)** | 1.54 (0.88-2.69) | **2.74 (1.54-4.87)** |  |
|  | Model 4 HR^f^ (95 % CI) | 1.00 | **1.95 (1.01-3.79)** | 1.46 (0.83-2.55) | **2.49 (1.40-4.43)** |  |
|  |  |  |  |  |  |  |
|  |  |  |  |  |  |  |
|  |  |  |  |  |  |  |
|  |  |  |  |  |  |  |
|  |  |  |  |  |  |  |
|  | **Pancreas (one year)** |  |  |  |  |  |
|  |  | MHNW | MHO | MUNW | MUHO |  |
|  | Participants | 30099 | 6377 | 11384 | 5182 |  |
|  | Parson-years | 246204.1 | 53399.4 | 91532.3 | 42344.0 |  |
|  | No. of cases | 37 | 10 | 39 | 18 |  |
|  | Model 1 HR^a^ (95 % CI) | 1.00 | 1.26 (0.62-2.55) | **1.69 (1.07-2.67)** | **1.89 (1.07-3.35)** |  |
|  | Model 2 HR^b^ (95 % CI) | 1.00 | 1.25 (0.62-2.52) | **1.68 (1.06-2.66)** | **1.90 (1.07-3.38)** |  |
|  | Model 3 HR^c^ (95 % CI) | 1.00 | 1.27 (0.63-2.57) | **1.70 (1.07-2.69)** | **1.96 (1.10-3.48)** |  |
|  |  | |  |  |  |  |
|  |  | |  |  |  |  |
|  | **Lung and Bronchus (one year)** | |  |  |  |  |
|  |  | MHNW | MHO | MUNW | MUHO |  |
|  | Participants | 30099 | 6377 | 11384 | 5182 |  |
|  | Parson-years | 246204.1 | 53399.4 | 91532.3 | 42344.0 |  |
|  | No. of cases | 145 | 31 | 87 | 38 |  |
|  | Model 1 HR^a^ (95 % CI) | 1.00 | 0.92 (0.62-1.36) | 0.99 (0.76-1.30) | 0.96 (0.67-1.39) |  |
|  | Model 2 HR^b^ (95 % CI) | 1.00 | 0.88 (0.59-1.30) | 1.00 (0.76-1.31) | 0.92 (0.64-1.33) |  |
|  | Model 3 HR^c^ (95 % CI) | 1.00 | 0.88 (0.59-1.30) | 1.00 (0.76-1.31) | 0.93 (0.65-1.34) |  |
|  | **Lung and Bronchus (two years)** | |  |  |  |  |
|  |  | MHNW | MHO | MUNW | MUHO |  |
|  | Participants | 30099 | 6377 | 11384 | 5182 |  |
|  | Parson-years | 246204.1 | 53399.4 | 91532.3 | 42344.0 |  |
|  | No. of cases | 131 | 28 | 80 | 36 |  |
|  | Model 1 HR^a^ (95 % CI) | 1.00 | 0.92 (0.61-1.39) | 1.01 (0.76-1.35) | 1.02 (0.70-1.48) |  |
|  | Model 2 HR^b^ (95 % CI) | 1.00 | 0.88 (0.58-1.33) | 1.02 (0.77-1.36) | 0.97 (0.67-1.41) |  |
|  | Model 3 HR^c^ (95 % CI) | 1.00 | 0.88 (0.58-1.33) | 1.02 (0.77-1.36) | 0.98 (0.67-1.42) |  |
|  | **Male** |  |  |  |  |  |
|  | **Total　(one year)** |  |  |  |  |  |
|  |  | MHNW | MHO | MUNW | MUHO |  |
|  | Participants | 11083 | 3597 | 5434 | 3130 |  |
|  | Parson-years | 89738.8 | 29773.1 | 42650.9 | 25117.7 |  |
|  | No. of cases | 743 | 205 | 547 | 302 |  |
|  | Model 1 HR^d^ (95 % CI) | 1.00 | 0.97 (0.83-1.14) | 1.09 (0.98-1.22) | **1.20 (1.05-1.37)** |  |
|  | Model 2 HR^b^ (95 % CI) | 1.00 | 0.95 (0.81-1.11) | 1.08 (0.97-1.21) | **1.16 (1.01-1.33)** |  |
|  | Model 3 HR^c^ (95 % CI) | 1.00 | 0.95 (0.81-1.11) | 1.08 (0.97-1.21) | **1.16 (1.01-1.32)** |  |
|  | **Total　(two years)** |  |  |  |  |  |
|  |  | MHNW | MHO | MUNW | MUHO |  |
|  | Participants | 11083 | 3597 | 5434 | 3130 |  |
|  | Parson-years | 89738.8 | 29773.1 | 42650.9 | 25117.7 |  |
|  | No. of cases | 658 | 189 | 490 | 270 |  |
|  | Model 1 HR^d^ (95 % CI) | 1.00 | 1.01 (0.86-1.19) | 1.12 (0.99-1.26) | **1.21 (1.05-1.40)** |  |
|  | Model 2 HR^b^ (95 % CI) | 1.00 | 0.98 (0.84-1.16) | 1.11 (0.98-1.24) | **1.18 (1.02-1.36)** |  |
|  | Model 3 HR^c^ (95 % CI) | 1.00 | 0.98 (0.83-1.16) | 1.11 (0.98-1.24) | **1.17 (1.02-1.35)** |  |
|  |  |  |  |  |  |  |
|  |  |  |  |  |  |  |
|  |  |  |  |  |  |  |
|  |  |  |  |  |  |  |
|  |  |  |  |  |  |  |
|  |  |  |  |  |  |  |
|  | **Prostate (one year)** |  |  |  |  |  |
|  |  | MHNW | MHO | MUNW | MUHO |  |
|  | Participants | 11083 | 3597 | 5434 | 3130 |  |
|  | Parson-years | 89738.8 | 29773.1 | 42650.9 | 25117.7 |  |
|  | No. of cases | 181 | 33 | 123 | 74 |  |
|  | Model 1 HR^d^ (95 % CI) | 1.00 | 0.69 (0.48-1.01) | 0.94 (0.74-1.18) | 1.21 (0.92-1.58) |  |
|  | Model 2 HR^b^ (95 % CI) | 1.00 | 0.70 (0.49-1.02) | 0.93 (0.74-1.17) | 1.22 (0.93-1.60) |  |
|  | Model 3 HR^c^ (95 % CI) | 1.00 | 0.70 (0.48-1.02) | 0.93 (0.74-1.18) | 1.21 (0.92-1.59) |  |
|  | **Prostate (two years)** |  |  |  |  |  |
|  |  | MHNW | MHO | MUNW | MUHO |  |
|  | Participants | 11083 | 3597 | 5434 | 3130 |  |
|  | Parson-years | 89738.8 | 29773.1 | 42650.9 | 25117.7 |  |
|  | No. of cases | 163 | 31 | 110 | 66 |  |
|  | Model 1 HR^d^ (95 % CI) | 1.00 | 0.71 (0.49-1.05) | 0.94 (0.74-1.20) | 1.19 (0.90-1.59) |  |
|  | Model 2 HR^b^ (95 % CI) | 1.00 | 0.73 (0.50-1.07) | 0.93 (0.73-1.19) | 1.22 (0.91-1.62) |  |
|  | Model 3 HR^c^ (95 % CI) | 1.00 | 0.73 (0.49-1.07) | 0.94 (0.74-1.20) | 1.21 (0.91-1.62) |  |
|  |  |  |  |  |  |  |
|  | **Female** |  |  |  |  |  |
|  | **Total (one year)** |  |  |  |  |  |
|  |  | MHNW | MHO | MUNW | MUHO |  |
|  | Participants | 19016 | 2780 | 5950 | 2052 |  |
|  | Parson-years | 156465.3 | 23626.2 | 48881.4 | 17226.3 |  |
|  | No. of cases | 791 | 159 | 353 | 139 |  |
|  | Model 1 HR^d^ (95 % CI) | 1.00 | **1.33 (1.12-1.58)** | 1.13 (0.99-1.29) | **1.31 (1.08-1.57)** |  |
|  | Model 2 HR^b^ (95 % CI) | 1.00 | **1.33 (1.12-1.58)** | 1.13 (0.99-1.29) | **1.31 (1.09-1.58)** |  |
|  | Model 3 HR^c^ (95 % CI) | 1.00 | **1.33 (1.12-1.58)** | 1.13 (0.99-1.29) | **1.31 (1.09-1.58)** |  |
|  | **Total (two years)** |  |  |  |  |  |
|  |  | MHNW | MHO | MUNW | MUHO |  |
|  | Participants | 19016 | 2780 | 5950 | 2052 |  |
|  | Parson-years | 156465.3 | 23626.2 | 48881.4 | 17226.3 |  |
|  | No. of cases | 699 | 139 | 313 | 124 |  |
|  | Model 1 HR^d^ (95 % CI) | 1.00 | **1.30 (1.08-1.56)** | 1.14 (0.99-1.31) | **1.31 (1.07-1.59)** |  |
|  | Model 2 HR^b^ (95 % CI) | 1.00 | **1.30 (1.08-1.56)** | 1.14 (0.99-1.32) | **1.31 (1.08-1.60)** |  |
|  | Model 3 HR^c^ (95 % CI) | 1.00 | **1.30 (1.08-1.56)** | 1.14 (0.99-1.31) | **1.31 (1.08-1.60)** |  |
|  | **Breast (one year)** |  |  |  |  |  |
|  |  | MHNW | MHO | MUNW | MUHO |  |
|  | Participants | 19016 | 2780 | 5950 | 2052 |  |
|  | Parson-years | 156465.3 | 23626.2 | 48881.4 | 17226.3 |  |
|  | No. of cases | 243 | 51 | 92 | 41 |  |
|  | Model 1 HR^d^ (95 % CI) | 1.00 | **1.41 (1.04-1.92)** | 1.20 (0.93-1.55) | **1.52 (1.08-2.14)** |  |
|  | Model 2 HR^b^ (95 % CI) | 1.00 | **1.41 (1.04-1.91)** | 1.21 (0.94-1.56) | **1.54 (1.09-2.17)** |  |
|  | Model 3 HR^c^ (95 % CI) | 1.00 | **1.42 (1.05-1.93)** | 1.21 (0.94-1.56) | **1.55 (1.10-2.18)** |  |
|  | Model 4 HR^g^ (95 % CI) | 1.00 | **1.42 (1.05-1.94)** | 1.20 (0.93-1.55) | **1.53 (1.09-2.16)** |  |
|  |  |  |  |  |  |  |
|  |  |  |  |  |  |  |
|  |  |  |  |  |  |  |
|  |  |  |  |  |  |  |
|  |  |  |  |  |  |  |
|  |  |  |  |  |  |  |
|  | **Breast (two years)** |  |  |  |  |  |
|  |  | MHNW | MHO | MUNW | MUHO |  |
|  | Participants | 19016 | 2780 | 5950 | 2052 |  |
|  | Parson-years | 156465.3 | 23626.2 | 48881.4 | 17226.3 |  |
|  | No. of cases | 214 | 44 | 82 | 34 |  |
|  | Model 1 HR^d^ (95 % CI) | 1.00 | 1.37 (0.98-1.90) | 1.23 (0.94-1.61) | 1.43 (0.98-2.08) |  |
|  | Model 2 HR^b^ (95 % CI) | 1.00 | 1.36 (0.98-1.89) | 1.24 (0.94-1.62) | 1.45 (0.99-2.10) |  |
|  | Model 3 HR^c^ (95 % CI) | 1.00 | 1.37 (0.99-1.91) | 1.23 (0.94-1.62) | 1.45 (1.00-2.11) |  |
|  | Model 4 HR^h^ (95 % CI) | 1.00 | 1.37 (0.98-1.91) | 1.22 (0.93-1.61) | 1.44 (0.99-2.09) |  |
|  | **Corpus uteri (one year)** |  |  |  |  |  |
|  |  | MHNW | MHO | MUNW | MUHO |  |
|  | Participants | 19016 | 2780 | 5950 | 2052 |  |
|  | Parson-years | 156465.3 | 23626.2 | 48881.4 | 17226.3 |  |
|  | No. of cases | 50 | 10 | 10 | 11 |  |
|  | Model 1 HR^d^ (95 % CI) | 1.00 | 1.47 (0.74-2.91) | 0.58 (0.29-1.18) | **2.07 (1.05-4.08)** |  |
|  | Model 2 HR^b^ (95 % CI) | 1.00 | 1.48 (0.75-2.94) | 0.58 (0.29-1.19) | **2.11 (1.06-4.17)** |  |
|  | Model 3 HR^c^ (95 % CI) | 1.00 | 1.48 (0.74-2.93) | 0.58 (0.29-1.18) | **2.09 (1.05-4.15)** |  |
|  | Model 4 HR^h^ (95 % CI) | 1.00 | 1.46 (0.74-2.92) | 0.59 (0.29-1.20) | **2.12 (1.07-4.22)** |  |
|  | HR, hazard ratio; CI, confidence interval; MHNW, Metabolically healthy normal weight; MUNW, Metabolically unhealthy normal weight; MHO  Metabolically healthy obesity; MUHO, Metabolically unhealthy obesity. | | | | | |
|  | ^a^ Adjusted for age, sex, research sites, and educational background. | | | | | |
|  | ^b^ Additionally adjusted for pack-years (four categories), drinking habit (four categories), and physical activity level (quartiles). | | | | | |
|  | ^c^ Additionally adjusted for miso soup (quartiles), fruits (quartiles), and vegetables (quartiles) consumption. | | | | | |
|  | ^d^ Adjusted for age, research sites, and educational background. | | | | | |
|  | ^e^ Additionally adjusted for antipyretic use (two categories), calcium (quartiles) , red meat (quartiles), and, processed meat (quartiles) consumption. | | | | | |
|  | ^f^ Additionally adjusted for history of hepatitis B (two categories) and hepatitis C (two categories). | | | | | |
|  | ^g^ Additionally adjusted for hormone replacement therapy (two categories), age of menarche (four categories), and age of menopause (five categories). | | | | | |
|  | ^h^ Additionally adjusted for hormone replacement therapy (two categories), age of menarche (four categories), age of menopause (five categories), and history of ovarian disease (three categories). | | | | | |

| **Supplementary Table 12. Multivariable hazard ratios and 95% confidence intervals for the association between metabolic phenotypes and breast cancer stratified by menopausal status.** | | | | | | |
| --- | --- | --- | --- | --- | --- | --- |
|  |  |  |  |  |  |  |
| **Breast (age ≤ 54)** |  |  |  |  |  |  |
|  | MHNW | MHO | MUNW | MUHO |  |  |
| Participants | 11742 | 1585 | 1263 | 511 |  |  |
| Parson-years | 96338.1 | 13462.3 | 10732.7 | 4392.8 |  |  |
| No. of cases | 245 | 37 | 25 | 18 |  |  |
| Model 1 HR^a^ (95 % CI) | 1.00 | 1.24 (0.87-1.76) | 0.95 (0.62-1.44) | **1.79 (1.10-2.91)** |  |  |
| Model 2 HR^b^ (95 % CI) | 1.00 | 1.22 (0.86-1.73) | 0.94 (0.62-1.42) | **1.79 (1.10-2.90)** |  |  |
| Model 3 HR^c^ (95 % CI) | 1.00 | 1.23 (0.87-1.75) | 0.94 (0.62-1.43) | **1.84 (1.13-3.00)** |  |  |
| Model 4 HR^d^ (95 % CI) | 1.00 | 1.23 (0.87-1.75) | 0.94 (0.62-1.43) | **1.86 (1.14-3.04)** |  |  |
| **Breast (age >54)** |  |  |  |  |  |  |
|  | MHNW | MHO | MUNW | MUHO |  |  |
| Participants | 7274 | 1195 | 4687 | 1541 |  |  |
| Parson-years | 76474.6 | 12420.8 | 41962.7 | 14112.2 |  |  |
| No. of cases | 125 | 34 | 96 | 41 |  |  |
| Model 1 HR^a^ (95 % CI) | 1.00 | **1.76 (1.20-2.58)** | 1.21 (0.92-1.58) | **1.63 (1.14-2.33)** |  |  |
| Model 2 HR^b^ (95 % CI) | 1.00 | **1.78 (1.21-2.62)** | 1.22 (0.93-1.59) | **1.66 (1.16-2.38)** |  |  |
| Model 3 HR^c^ (95 % CI) | 1.00 | **1.78 (1.21-2.62)** | 1.20 (0.92-1.58) | **1.64 (1.14-2.35)** |  |  |
| Model 4 HR^d^ (95 % CI) | 1.00 | **1.78 (1.21-2.61)** | 1.20 (0.91-1.57) | **1.63 (1.14-2.33)** |  |  |
| HR, hazard ratio; CI, confidence interval; MHNW, Metabolically healthy normal weight; MUNW, Metabolically unhealthy normal weight; MHO Metabolically healthy obesity;  MUHO, Metabolically unhealthy obesity. | | | | | | |
| ^a^ Adjusted for age, research sites, and educational background. | | | | | |  |
| ^b^ Additionally adjusted for pack-years (four categories), drinking habit (four categories), and physical activity level (quartiles). | | | | | |  |
| ^c^ Additionally adjusted for miso soup (quartiles), fruits (quartiles), and vegetables (quartiles) consumption. | | | | | |  |
| ^d^ Additionally adjusted for hormone replacement therapy (two categories), age of menarche (four categories). | | | | | |  |

| **Supplementary Table 13. Multivariable hazard ratios and 95% confidence intervals for the association between metabolic phenotypes and proximal and distal colon cancer and rectum cancer.** | | | | |
| --- | --- | --- | --- | --- |
| **Proximal colon cancer** |  |  |  |  |
|  | MHNW | MHO | MUNW | MUHO |
| Participants | 30099 | 6377 | 11384 | 5182 |
| Parson-years | 246204.1 | 53399.4 | 91532.3 | 42344.0 |
| No. of cases | 72 | 23 | 55 | 25 |
| Model 1 HR^a^ (95 % CI) | 1.00 | 1.57 (0.97-2.52) | 1.27 (0.89-1.82) | 1.43 (0.90-2.28) |
| Model 2 HR^b^ (95 % CI) | 1.00 | 1.58 (0.98-2.54) | 1.26 (0.88-1.81) | 1.44 (0.90-2.28) |
| Model 3 HR^c^ (95 % CI) | 1.00 | 1.56 (0.97-2.51) | 1.25 (0.87-1.80) | 1.42 (0.89-2.25) |
| Model 4 HR^d^ (95 % CI) | 1.00 | 1.57 (0.98-2.53) | 1.25 (0.87-1.80) | 1.42 (0.89-2.26) |
| **Distal colon cancer** |  |  |  |  |
|  | MHNW | MHO | MUNW | MUHO |
| Participants | 30099 | 6377 | 11384 | 5182 |
| Parson-years | 246204.1 | 53399.4 | 91532.3 | 42344.0 |
| No. of cases | 76 | 28 | 40 | 27 |
| Model 1 HR^a^ (95 % CI) | 1.00 | **1.66 (1.07-2.58)** | 1.00 (0.68-1.49) | 1.54 (0.98-2.41) |
| Model 2 HR^b^ (95 % CI) | 1.00 | **1.66 (1.07-2.58)** | 1.00 (0.68-1.49) | 1.52 (0.97-2.38) |
| Model 3 HR^c^ (95 % CI) | 1.00 | **1.65 (1.06-2.56)** | 1.00 (0.67-1.48) | 1.50 (0.96-2.36) |
| Model 4 HR^d^ (95 % CI) | 1.00 | **1.64 (1.06-2.55)** | 0.99 (0.67-1.47) | 1.47 (0.94-2.32) |
|  |  |  |  |  |
| **Rectum cancer** |  |  |  |  |
|  | MHNW | MHO | MUNW | MUHO |
| Participants | 30099 | 6377 | 11384 | 5182 |
| Parson-years | 246204.1 | 53399.4 | 91532.3 | 42344.0 |
| No. of cases | 104 | 25 | 62 | 37 |
| Model 1 HR^a^ (95 % CI) | 1.00 | 1.08 (0.70-1.68) | 1.05 (0.76-1.45) | 1.47 (1.00-2.15) |
| Model 2 HR^b^ (95 % CI) | 1.00 | 1.08 (0.70-1.68) | 1.03 (0.75-1.43) | 1.42 (0.97-2.08) |
| Model 3 HR^c^ (95 % CI) | 1.00 | 1.09 (0.70-1.70) | 1.04 (0.75-1.44) | 1.43 (0.97-2.10) |
| Model 4 HR^d^ (95 % CI) | 1.00 | 1.09 (0.70-1.69) | 1.04 (0.75-1.44) | 1.42 (0.96-2.08) |
| HR, hazard ratio; CI, confidence interval; MHNW, Metabolically healthy normal weight; MUNW, Metabolically unhealthy normal weight; MHO Metabolically healthy obesity; MUHO, Metabolically unhealthy obesity. | | | | |
| ^a^ Adjusted for age, sex, research sites, and educational background. | | | | |
| ^b^ Additionally adjusted for pack-years (four categories), drinking habit (four categories), and physical activity level (quartiles). | | | | |
| ^c^ Additionally adjusted for miso soup (quartiles), fruits (quartiles), and vegetables (quartiles) consumption. | | | | |
| ^d^ Additionally adjusted for antipyretic use (two categories), calcium (quartiles) , red meat (quartiles), and, processed meat (quartiles) consumption. | | | | |

| **Supplementary Table 14.** The incident cases of site-specific cancer according to sex and obesity. | | | | | | |
| --- | --- | --- | --- | --- | --- | --- |
| Examination-based analyses |  |  |  |  |  |  |
| Organs | Male | | Organs | Female | |  |
|  | Normal Weight (n=8657) | Obesity  (n=3812) |  | Normal Weight (n=10391) | Obesity  (n=2497) |  |
|  |  |  |  |  |  |  |
| Stomach (C16) | 121 | 44 | Stomach (C16) | 52 | 10 |  |
| Colon and rectum (C18-21) | 81 | 47 | Colon and rectum (C18-21) | 65 | 20 |  |
| Bronchus, lung (C34) | 71 | 23 | Bronchus, lung (C34) | 37 | 12 |  |
| Prostate (C61) | 162 | 55 | Breast (C50) | 124 | 51 |  |
|  |  |  | Female genital cancer (C51-C58) | 70 | 15 |  |
| Others | 225 | 122 | Others | 127 | 50 |  |
| Total | 660 | 291 | Total | 475 | 158 |  |
|  |  |  |  |  |  |  |
| Questionnaire-based analyses |  |  |  |  |  |  |
| Organs | Male | | Organs | Female | |  |
|  | Normal Weight (n=16517) | Obesity  (n=6727) |  | Normal Weight (n=24966) | Obesity  (n=4832) |  |
|  |  |  |  |  |  |  |
| Stomach (C16) | 301 | 91 | Stomach (C16) | 130 | 23 |  |
| Colon and rectum (C18-21) | 230 | 108 | Colon and rectum (C18-21) | 187 | 62 |  |
| Liver (C22) | 50 | 37 | Liver (C22) | 24 | 13 |  |
| Pancreas (C25) | 80 | 23 | Pancreas (C25) | 47 | 15 |  |
| Bronchus, lung (C34) | 231 | 87 | Bronchus, lung (C34) | 140 | 28 |  |
| Prostate (C61) | 359 | 129 | Breast (C50) | 491 | 130 |  |
|  |  |  | Corpus uteri (C54) | 82 | 24 |  |
| Others | 506 | 191 | Others | 536 | 112 |  |
| Total | 1757 | 666 | Total | 1637 | 407 |  |
|  |  |  |  |  |  |  |
