# Supplementary material for: The Significance of Comprehensive Metabolic Phenotypes in Cancer Risk: A Japan Multi-Institutional Collaborative Cohort Study
Source: Cancer Res Commun. 2024 Nov 21;4(11):2986–97. doi: 10.1158/2767-9764.CRC-24-0249 (PMC11579844; doi:10.1158/2767-9764.CRC-24-0249)
Supplement: Supplementary Data — Supplementary File (List of the contributors to the J-MICC Study) [file crc-24-0249_supplementary_data_suppsd.docx]

**List of the contributors to the J-MICC Study (**<https://jmicc.com/en/contributors>.**)**

**Chiba Cancer Center**

Nobuaki Michihata
Yohko Nakamura
Haruo Mikami
Hiroki Nagase
Kimiko Takayama

**Kanagawa Cancer Center**

Hiroto Narimatsu
Sho Nakamura
Kaname Watanabe
Kayoko Katayama
Yoshinobu Saito
Ryoko Kawakami
CHEI CHOY LYE
Masumi Okamoto
Naoko Shinmura
Koshi Takahashi

**Shizuoka Prefectual University**

Kiyonori Kuriki

**Nagoya City University**

Sadao Suzuki
Takeshi Nishiyama
Takahiro Otani
Hiroko Nakagawa
Shinkan Tokudome
Akihiro Hosono
Yuya Tamai
Miki Watanabe
Makoto Sunayama

**Aichi Cancer Center**

Keitaro Matsuo
Hidemi Ito
Isao Oze
Yuriko Koyanagi
Yuji Iwashita
Yumiko Kasugai
Masanori Kawaura
Kazuo Tajima
Wakai Kenji
Kaoru Hirose
Akio Hiraki
Hideo Tanaka
Takeshi Suzuki
Takakazu Kawase
Satoyo Hosono
Yukari Taniyama

**Nagoya University**

Kenji Wakai
Takashi Tamura
Mako Nagayoshi
Takashi Matsunaga
Rieko Okada
Yoko Kubo
Yoko Mitsuda
Yasufumi Kato
Hidemi Hattori
Masahiro Nakatochi
Yoshiko Ishida
Satoshi Osafune
Yuka Kadomatsu
Sayo Kawai
Etsuko Kimura
Sayaka Kuriki
Tae Sasakabe
Yuka Sugimoto
Shino Suma
Toshio Seiki
Sahoko Takagi
Kenji Takeuchi
Akiko Tamakoshi
Yudai Tamada
Mineko Tsukamoto
TowaTai（Yin Guang）
Kotaro Tomita
Mariko Naito
Hiroko Nakagawa
Kazuko Nishio
Yuta Hattori
Nobuyuki Hamajima
Takahiro Higashibata
Asahi Hishida
Nana Fukuda
Kaori Masui
Kenta Maruyama
Keiko Mizutani
Emi Morita

**Shiga Medical University/Tsuruga Nursing University**

Kuniyoshi Kita
Katsuyuki Miura
Yasuyuki Nakamura
Naotaka Takashima
Kenji Matsui
Aya Kadota
Tanvir Chowdhry Turin
Naoko Miyagawa
Hiroshi Ueshima
Fusako Katsurada
Masae Torii
Etsuko Maekawa

**Kyoto Prefectual University of Medicine**

Teruhide Koyama
Etsuko Ozaki
Naoyuki Takashima
Daisuke Matsui
Isao Watanabe
Reo Nagamitsu
Yoshiyuki Watanabe
Ritei Uehara
Nagato Kuriyama
Satomi Tomida
Yukiko Nukaya
Mizuho Wada
Komei Iwai
Chie Omichi
Rika Tanaka
Fumitaro Miyatani
Mao Hirota
Aya Yoshikawa
Kumiko Hara
Satoko Mitani

**Tokushima University**

Kokichi Arisawa
Sakurako Kamano
Hirokazu Uemura
Miwa Yamaguchi
Mineyoshi Hiyoshi
Mariko Nakamoto
Masashi Ishizu
Takeshi Watanabe
Noriko Tsuruta
Natsuko Yamamoto
Momoko Yamaguchi
Manami Inohara
Rie Matsumura
Yayoi Asano
Hidenobu Takami
Tirani Bahari
Yuki Iwasaki
Tien Van Nguyen

**Kyushu University**

Jun Hayashi
Norihiro Furusho
Hiroaki Ikezaki
Suminori Kono
Keizo Ohnaka
Makiko Umemoto
Ryoko Nakashima

**Saga University**

Keitaro Tanaka
Megumi Hara
Yuichiro Nishida
Chisato Shimanoe
Takuma Furukawa
Hinako Nanri
Yasuki Higaki
Koichi Shinchi

**Kagoshima University**

Chihaya Koriyama
Shiro Tanoue
Toshiro Takezaki
Hideshi Niimura
Rie Ibusuki
Kazuyo Kuwabara
Noriko Nakahata
Masaya Tatebo
Keiichi Shimatani
Ippei Shimoshikiryo
Daisaku Nishimoto
Kenichi Shibuya
Rika Matsuyama
Chiharu Takada
Yoshifumi Hidaka
Motahareh Kheradmand
Eva Mariane Mantjoro
Tara Sefanya Kairupan
Yora Nindita
Athira Dhruva
Saekhol Bakri
Inria Astari Zahra

**Aichi Medical University**

Asahi Hishida

**Research Supporting Group**

Kenji Wakai  
Hidetaka Eguchi
Naomi Imaeda
Chiho Goto
Yukihide Tomozawa
Tomohiro Shinozaki
Shuji Hashimoto
Takashi Takahashi
Akihiro Sekine
Kei Nakachi
Kazue Imai
Michiaki Kubo
